# PFKM Modulates Porcine Skeletal Muscle Satellite Cell Differentiation Through Metabolic and Mitochondrial Pathways

**DOI:** 10.3390/vetsci13080742

**Published:** 2026-07-26

**Authors:** Xiaoyu Hou, Yuefei Yang, Ruiping Wei, Xiaochen Cui, Huanyu Jiang, Huiming Ju

**Affiliations:** 1 Jiangsu Co-Innovation Center for Prevention and Control of Important Animal Infectious Diseases and Zoonoses, Yangzhou University, Yangzhou 225009, China; 17863660983@163.com (X.H.); yyf7607@163.com (Y.Y.);; 2 Laboratory of Lingnan Modern Agriculture, Key Laboratory of Livestock and Poultry Multi-Omics of MARA, Agricultural Genomics Institute at Shenzhen, Chinese Academy of Agricultural Sciences, Shenzhen 518124, China; gabrielwrp0920@gmail.com

**Keywords:** muscle-type phosphofructokinase, pig, skeletal muscle satellite cells, myogenic differentiation, cellular metabolism

## Abstract

To preliminarily elucidate the bidirectional metabolic effects caused by changes in PFKM expression, provide research clues for further investigation of the molecular mechanisms through which PFKM regulates porcine skeletal muscle metabolism and myogenic differentiation, and offer a reference for identifying candidate genes associated with meat production traits and studying gene regulation in pig breeding, this study compared skeletal muscle protein expression profiles between Large White and Bama pigs. Candidate regulatory factors related to muscle growth and energy metabolism were screened, and the effects of altered muscle-type phosphofructokinase (PFKM) expression on metabolic homeostasis and myogenic differentiation in porcine skeletal muscle satellite cells (SMSCs) were preliminarily evaluated. Longissimus dorsi muscle tissues from Large White and Bama pigs were analyzed using iTRAQ-based proteomics. A total of 2040 reliably quantified proteins were identified, of which 51 were relatively upregulated in Large White pigs and 73 were relatively upregulated in Bama pigs. Functional enrichment analysis showed that the differentially expressed proteins were mainly involved in glycolysis, mitochondrial energy metabolism, protein synthesis, and the regulation of muscle fiber structure and function. PFKM was therefore selected as a key candidate differentially expressed protein. Porcine SMSC models comprising a PFKM knockdown group (PFKM-KD), a PFKM overexpression group (PFKM-OE), and a normal control group (PFKM-CON) were subsequently established. Glucose consumption and lactate accumulation in the culture medium, ATP levels, reactive oxygen species (ROS), mitochondrial membrane potential, apoptosis, mitochondrial dynamics-related proteins, and myogenic differentiation markers were then examined. Compared with the PFKM-CON group, the PFKM-OE group showed significantly increased glucose consumption and lactate accumulation, together with significant increases in ROS levels, mitochondrial membrane potential, and apoptosis, whereas ATP levels were significantly reduced. In the PFKM-KD group, glucose consumption and lactate accumulation were significantly decreased, accompanied by reductions in mitochondrial membrane potential, ROS, ATP levels, and apoptosis. PFKM overexpression mainly induced oxidative stress, ATP depletion, and increased apoptosis, whereas PFKM knockdown primarily reduced mitochondrial membrane potential, ROS, and ATP levels, indicating a relatively low-metabolic state. Both treatments were accompanied by dysregulated expression of the mitochondrial dynamics-related proteins DRP1, MFN2, and OPA1, although their patterns of change were not identical. Western blotting and immunofluorescence consistently showed that the expression levels of the myogenic differentiation markers MyoD and MYH were significantly lower in both the PFKM-KD and PFKM-OE groups than in the control group, suggesting that either excessive or insufficient PFKM expression may impair the myogenic differentiation potential of SMSCs. In conclusion, changes in PFKM expression are closely associated with glycolysis-related metabolism, energy and redox homeostasis, mitochondrial function-related indicators, and myogenic differentiation capacity in porcine SMSCs. The normal biological function of PFKM may therefore depend on its expression being maintained within an appropriate range.

## Simple Summary

This study compared Large White and Bama pigs to identify differentially expressed skeletal muscle proteins using iTRAQ proteomics, with the glycolytic rate-limiting enzyme PFKM selected as a key candidate. Porcine skeletal muscle satellite cell models with PFKM overexpression or knockdown were then established to evaluate changes in glycolytic metabolism, ATP production, reactive oxygen species, mitochondrial membrane potential, apoptosis, and myogenic differentiation. PFKM overexpression mainly induced oxidative stress, ATP depletion, and increased apoptosis, whereas PFKM knockdown reduced mitochondrial membrane potential, ROS, and ATP levels, indicating a relatively low-metabolic state. Both treatments decreased MyoD and MYH expression, suggesting that either excessive or insufficient PFKM expression impairs myogenic differentiation. These findings provide a basis for further investigation of PFKM-related regulatory mechanisms and offer useful evidence for identifying candidate genes associated with pork production traits and studying gene regulation in pig breeding.

## 1. Introduction

The growth and development of porcine skeletal muscle directly affect carcass composition, meat production efficiency, and meat quality, and therefore represent a long-standing research focus in the field of animal genetics and breeding. The formation of the skeletal muscle phenotype is a complex biological process jointly influenced by genetic background, energy metabolism, cellular signal transduction, and environmental factors. Through tightly regulated spatiotemporal expression, multiple regulatory factors collectively participate in the activation, proliferation, migration, and differentiation of myogenic cells, as well as in the formation of muscle fiber structure. Although considerable progress has been made in understanding the regulation of skeletal muscle development in recent years, the molecular basis underlying the establishment of muscle growth and metabolic characteristics in different genetic backgrounds remains incompletely understood. The specific functions and mechanisms of many candidate regulatory factors also require further investigation [1,2].

Skeletal muscle satellite cells (SMSCs) are a population of adult myogenic stem cells located between the basal lamina and the sarcolemma of muscle fibers and were first identified by Mauro in 1961. Under physiological conditions, SMSCs generally remain quiescent. In response to muscle growth, injury, or changes in the metabolic environment, they can become activated and subsequently enter proliferation and differentiation programs. They contribute to skeletal muscle growth, repair, and regeneration by forming new muscle fibers or fusing with existing fibers. A reduction in satellite cell number or impairment of their function can markedly weaken the regenerative capacity of skeletal muscle [3,4]. During the maintenance of quiescence, activation, and myogenic differentiation, SMSCs undergo metabolic remodeling according to their functional demands and are exposed to multiple stimuli, including energetic stress, oxidative stress, and changes in mitochondrial homeostasis. These alterations may act as physiological signals that promote cellular adaptation and differentiation; however, once they exceed the compensatory capacity of the cells, they may lead to metabolic dysfunction, impaired differentiation, or even cell death [5,6]. Therefore, elucidating the relationship between the metabolic state of SMSCs and their myogenic fate is important for understanding the mechanisms of skeletal muscle growth and development and for identifying potential regulators of muscle-related traits [7,8].

Muscle-type phosphofructokinase (PFKM) is a key rate-limiting enzyme in the glycolytic pathway. It primarily uses adenosine triphosphate (ATP) as the phosphate donor to catalyze the conversion of fructose-6-phosphate to fructose-1,6-bisphosphate, with the concomitant production of adenosine diphosphate (ADP) [9,10]. Because this reaction occurs at a major regulatory step in glycolysis, changes in PFKM expression or enzymatic activity may affect glucose utilization, lactate production, energy supply, and the distribution of metabolic intermediates. Previous studies have shown that impaired PFKM function can reduce glucose utilization in skeletal muscle and cause decreased exercise tolerance, muscle damage, and related metabolic abnormalities [11]. In pigs, the PFKM gene is predominantly expressed in tissues with high energy demands, such as skeletal muscle and cardiac muscle. Genetic variation and differences in PFKM expression have also been reported among pig breeds, suggesting that PFKM may be associated with breed-specific characteristics of muscle growth and metabolism [12]. However, systematic evidence regarding how changes in PFKM expression affect metabolic homeostasis and myogenic differentiation in porcine SMSCs remains limited.

Large White pigs are a typical modern commercial lean-type breed characterized by a large body size, rapid growth, and a high lean meat percentage. In contrast, Bama pigs, a Chinese indigenous breed, differ markedly from Large White pigs in body size, growth rate, muscle fiber characteristics, and meat quality [13,14]. Differences in their genetic backgrounds and long-term selection histories make these two breeds a representative comparative model for investigating the molecular basis of skeletal muscle growth, energy metabolism, and meat quality formation. In our previous work, omics-based approaches were used to screen differentially expressed molecules in skeletal muscle tissues from different pig breeds. Multiple metabolism-related proteins, including PFKM, showed breed-dependent expression differences, suggesting that glycolysis and mitochondrial energy metabolism may contribute to the formation of distinct muscle phenotypes among pig breeds. Nevertheless, proteomic screening primarily reveals associations between candidate molecules and biological processes. Whether PFKM participates in the regulation of metabolism and myogenic differentiation in porcine SMSCs, and what cellular effects may result from either excessive or insufficient PFKM expression, still require further functional validation.

Therefore, in the present study, longissimus dorsi muscle tissues from Large White and Bama pigs were used to compare proteomic expression profiles and preliminarily identify differentially expressed proteins associated with skeletal muscle growth and energy metabolism. PFKM was selected as a key candidate regulatory factor. On this basis, porcine SMSC models with PFKM overexpression or knockdown were established, and changes in glucose consumption, lactate production, ATP levels, mitochondrial membrane potential, reactive oxygen species, apoptosis, mitochondrial dynamics-related proteins, and myogenic differentiation markers were examined. These analyses were performed to preliminarily evaluate the effects of dysregulated PFKM expression on glycolysis-related metabolism, cellular energy and redox homeostasis, mitochondrial status, and myogenic differentiation capacity. This study was designed to provide research clues and experimental directions for further elucidating the molecular mechanisms through which PFKM regulates porcine skeletal muscle metabolism and myogenic differentiation, and to offer a reference for identifying candidate genes associated with meat production traits and investigating gene regulation in pig genetic breeding.

## 2. Materials and Methods

### 2.1. iTRAQ Screening of Differentially Expressed Proteins in Skeletal Muscle Tissues from Different Pig Breeds

In this study, longissimus dorsi muscle samples were collected from 50-day-old Large White pigs and Bama pigs (three animals per breed). Total protein was extracted from skeletal muscle tissues, and protein concentrations were determined using the BCA (Bicinchoninic Acid) method. After reduction and alkylation, protein samples were digested with trypsin and labeled using iTRAQ 8-plex (Isobaric Tags for Relative and Absolute Quantitation, 8-plex) reagents. Data were obtained through high-performance liquid chromatography (HPLC) fractionation coupled with tandem mass spectrometry (LC-MS/MS). Subsequent protein identification and sequence analysis were performed by Shanghai Applied Protein Technology Co., Ltd., Shanghai, China. The experimental procedures and workflows followed the same principles as described in reference [15].

### 2.2. SMSCs Grouping and Treatments

**Figure 1:**
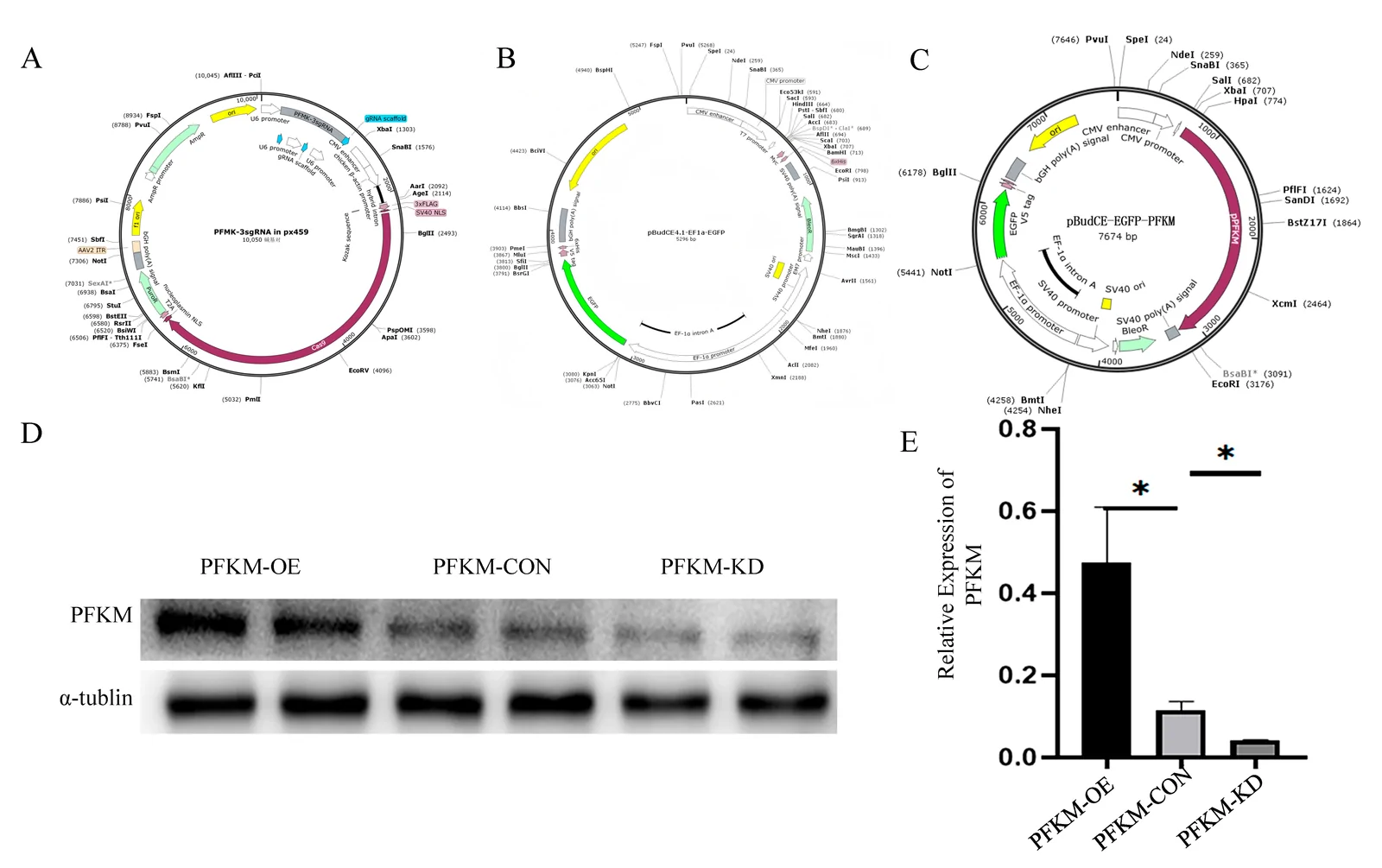
Schematic diagram for acquisition and identification of PFKM knockout plasmid and acquisition of PFKM knockdown and overexpression cell populations and detection of their efficiency. (**A**) Schematic diagram of PFKM gene knockdown vector. (**B**) Schematic diagram of blank control vector. (**C**) Schematic diagram of PFKM overexpression vector. (**D**) Detection of PFKM expression in cells. (**E**) Grayscale analysis of PFKM expression. * *p* < 0.05.

All experiments were conducted at the College of Veterinary Medicine, Yangzhou University. Porcine skeletal muscle satellite cells (SMSCs) isolated in our laboratory were divided into three groups. Cells transfected with the empty pBudCE-EGFP plasmid were used as the control group (PFKM-CON). Cells transfected with the pBudCE-EGFP-PFKM plasmid carrying the in vitro reverse-transcribed porcine PFKM sequence and subsequently selected with bleomycin to obtain a stably expressing cell population were designated as the PFKM overexpression group (PFKM-OE). Three single-guide RNAs (sgRNAs) were designed based on the Exon 8 sequence of the PFKM gene. The Exon 8 sequence was as follows: GTACCTGGCCCTTGTCACCTCTCTCTCCTGTGGGGCCGACTGGGTTTTTATTCCTGAGTGTCCGCCGGATGATGCCTGGGAGGAGCATCTTTGTCGCCGGCTCAGCGAG. The three underlined regions within this sequence were selected as the sgRNA target sequences. The CRISPR/Cas9 system was used to disrupt the PFKM gene in a proportion of the cells, followed by puromycin selection to obtain a stable cell population, which was designated as the PFKM knockdown group (PFKM-KD).Schematic diagrams of the corresponding vectors are shown in Figure 1 (Figure 1A–C), and the PFKM protein expression levels in each group are presented in Figure 1 (Figure 1D,E). Cell selection was performed according to the method described in reference [16].

### 2.3. Flow Cytometric Analysis of the Effects of Altered PFKM Expression on Mitochondrial Membrane Potential in SMSCs

Cells from each group were digested with EDTA-containing trypsin(Ethylenediaminetetraacetic acid Ethylenediaminetetraacetic acid) to prepare single-cell suspensions. The staining working solution was prepared according to the manufacturer’s instructions, and 1 mL of staining solution was added to each sample to resuspend, stain, and wash the cells. Detailed procedures were performed following the instructions provided with the mitochondrial membrane potential assay kit (Beyotime, Cat. No. C2006; Beyotime Biotechnology, Shanghai, China). Mitochondrial membrane potential levels in each group were analyzed using flow cytometry.

### 2.4. Analysis of the Effects of Altered PFKM Expression on Apoptosis in SMSCs

Cells from each group were digested with EDTA-containing trypsin to generate single-cell suspensions, followed by centrifugation at 800× *g* for 10 min. The supernatant was discarded, and 210 μL of staining solution was prepared per dish according to the manufacturer’s instructions. Cells were resuspended, stained, and washed as instructed in the apoptosis detection kit manual (Beyotime, C1062S). Apoptosis levels in PFKM-overexpressing and PFKM-knockdown SMSCs were analyzed by flow cytometry. Total protein was extracted from cells of each group. After separation by polyacrylamide gel electrophoresis and transfer to membranes, α-tubulin was used as the internal control (Proteintech, Cat. No. 11224-1-AP; Proteintech Group, Inc., Rosemont, IL, USA). The expression levels of apoptosis-related proteins Bcl-2 (B-cell lymphoma) (Santa Cruz Biotechnology, Cat. No. sc-783; Santa Cruz Biotechnology, Inc., Dallas, TX, USA) and BAX (Bcl-2 Associated X protein) (Santa Cruz Biotechnology, sc-6236) were detected by Western blotting. Band intensities were quantified using ImageJ software (Java 1.8.0). The relative expression levels of target proteins were calculated as the ratio of the gray value of the target band to that of the corresponding internal control band. Experimental procedures were performed as described in reference [17].

### 2.5. Effects of Altered PFKM Expression on ATP Levels in SMSCs

When the cell confluence reached approximately 90%, cells from each group were digested with EDTA-containing trypsin and centrifuged at 800× *g* for 10 min. After discarding the supernatant, detection reagents were diluted according to the manufacturer’s recommended ratios. For each sample, 100 μL of detection working solution was added to resuspend, stain, and wash the cells. Detailed procedures followed the instructions of the ATP assay kit (Beyotime, S0027). ATP levels in PFKM-overexpressing and PFKM-knockdown SMSCs were measured using a fluorescence microplate reader.

### 2.6. Flow Cytometric Analysis of the Effects of Altered PFKM Expression on Reactive Oxygen Species (ROS) Levels in SMSCs

Cells from each group were digested with EDTA-containing trypsin and centrifuged at 800× *g* for 10 min, after which the supernatant was discarded. DCFH-DA(2′,7′-Dichlorodihydrofluorescein diacetate) was diluted in PBS at a ratio of 1:2000, and 500 μL of the staining solution was added per dish to resuspend, stain, and wash the cells according to the manufacturer’s instructions. Experimental procedures followed the ROS detection kit manual (Beyotime, S0033S). Intracellular ROS levels in each group were determined by flow cytometry.

### 2.7. Western Blot Analysis of the Effects of Altered PFKM Expression on Mitochondrial Function–Related Protein Levels in SMSCs

Total protein was extracted from cells of each group, and protein concentrations were determined using the BCA method. With α-tubulin as the internal control, Western blot analysis was performed to detect the expression of mitochondrial function–related proteins MFN2 (Santa Cruz Biotechnology, sc-50331), OPA1 (NOVUS, Cat. No. NB110-55290; Novus Biologicals, LLC, Centennial, CO, USA), and DRP1 (Santa Cruz Biotechnology, sc-21804). Protein detection and data analysis were conducted using the same methods described in Section 2.4.

### 2.8. Analysis of the Effects of Altered PFKM Expression on Myogenic Differentiation of SMSCs

When cells in each group reached approximately 70% confluence, they were washed with PBS and then cultured in myogenic differentiation induction medium (DMEM (Dulbecco’s Modified Eagle Medium) high-glucose medium supplemented with 2% horse serum, 1% penicillin–streptomycin). The induction medium was replaced after 48 h, and cell growth and differentiation were monitored. Experimental procedures were performed as described in reference [18]. After myogenic induction, total cellular protein was extracted from the three groups, and protein concentrations were determined using the BCA method. With α-tubulin as the internal control, Western blot analysis was conducted to detect the expression of myogenic differentiation marker proteins MyoD (Santa Cruz Biotechnology, sc-377460) and MYH (Santa Cruz Biotechnology, sc-53088). Protein detection and data analysis were performed as described in Section 2.4. After 48 h of myogenic induction, the culture medium was removed, and cells were fixed with 4% paraformaldehyde and permeabilized with 0.5% Triton X-100. Following blocking with 2% BSA for 30 min, cells were incubated with antibodies against MYH (Santa Cruz Biotechnology, sc-53088) and MyoD (Santa Cruz Biotechnology, sc-377460) for immunofluorescence staining. All experiments were performed in triplicate. Fluorescence intensity was quantified using ImageJ software (https://imagej.net/, accessed on 21 July 2026). Detailed procedures followed those described in reference [7].

### 2.9. Analysis of the Effects of Altered PFKM Expression on Glycolytic Activity in SMSCs

Equal numbers of cells from the three groups were cultured for 72 h, after which the culture media were collected. According to the instructions of the glucose assay kit (Beyotime, S0201S) and lactate assay kit (Beyotime, S0208S), absorbance was measured at 620 nm and 450 nm, respectively, to determine glucose and lactate concentrations in the culture media. The glucose consumption and lactate accumulation of each group were calculated as the differences between the glucose and lactate levels in the conditioned media and those in complete culture medium incubated under the same conditions without cells.

### 2.10. Statistical Analysis

Differentially expressed proteins in the proteomic analysis were screened using an absolute fold change of ≥1.3 and a statistically significant difference between groups (*p* < 0.05) as the selection criteria. The protein lists identified in each sample were subjected to bioinformatics analyses, including Gene Ontology (GO) functional annotation and enrichment analysis and Kyoto Encyclopedia of Genes and Genomes (KEGG) pathway enrichment analysis. GO analysis of the differentially expressed proteins focused on molecular function (MF), biological process (BP), and cellular component (CC), whereas KEGG analysis focused particularly on pathways associated with metabolism and muscle fiber structure.

All data from gene functional experiments were statistically analyzed using GraphPad Prism 7 (https://www.graphpad.com/, accessed on 21 July 2026). Data are presented as the mean ± standard deviation ($\bar{x}$ ± SD). The PFKM normal control group (PFKM-CON), PFKM overexpression group (PFKM-OE), and PFKM knockdown group (PFKM-KD) were treated as three independent experimental groups, and each outcome variable was analyzed separately. Overall differences among the three groups were evaluated using ordinary one-way analysis of variance (one-way ANOVA). Dunnett’s multiple-comparisons test was subsequently performed to compare the PFKM-OE group with the PFKM-CON group and the PFKM-KD group with the PFKM-CON group. Flow cytometry, immunofluorescence, glucose consumption, lactate production, and other cellular functional assays were performed using three independent biological samples. The current Western blot experiments included two independent samples per group. Therefore, the corresponding means and standard deviations can only provide a preliminary indication of the observed trends, and the robustness of the statistical inference is limited. All statistical tests were two-sided. A value of *p* < 0.05 was considered statistically significant. Statistical significance in the figures was generally indicated as follows: * *p* < 0.05.

## 3. Results

### 3.1. Screening of Differentially Expressed Proteins in Skeletal Muscle Tissues from Different Pig Breeds

**Figure 2:**
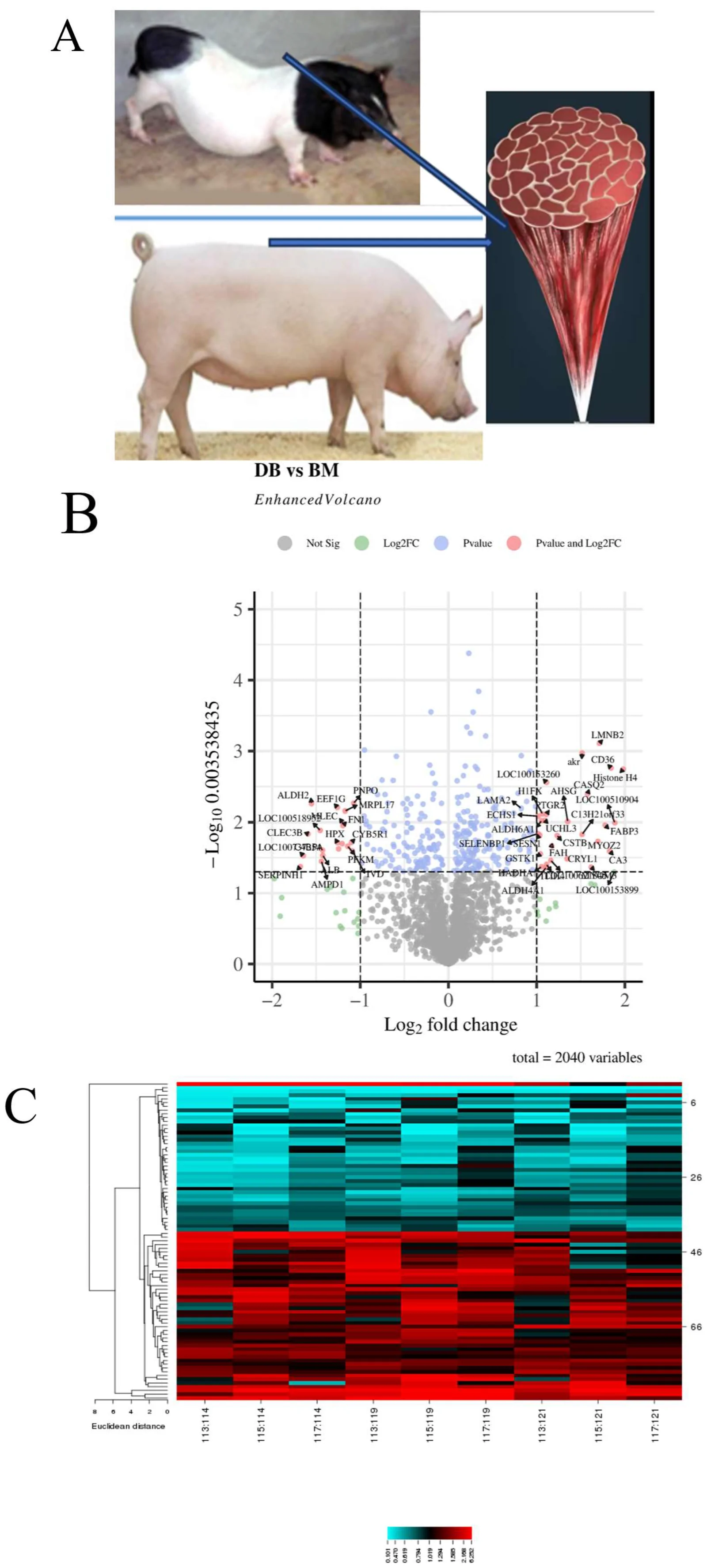

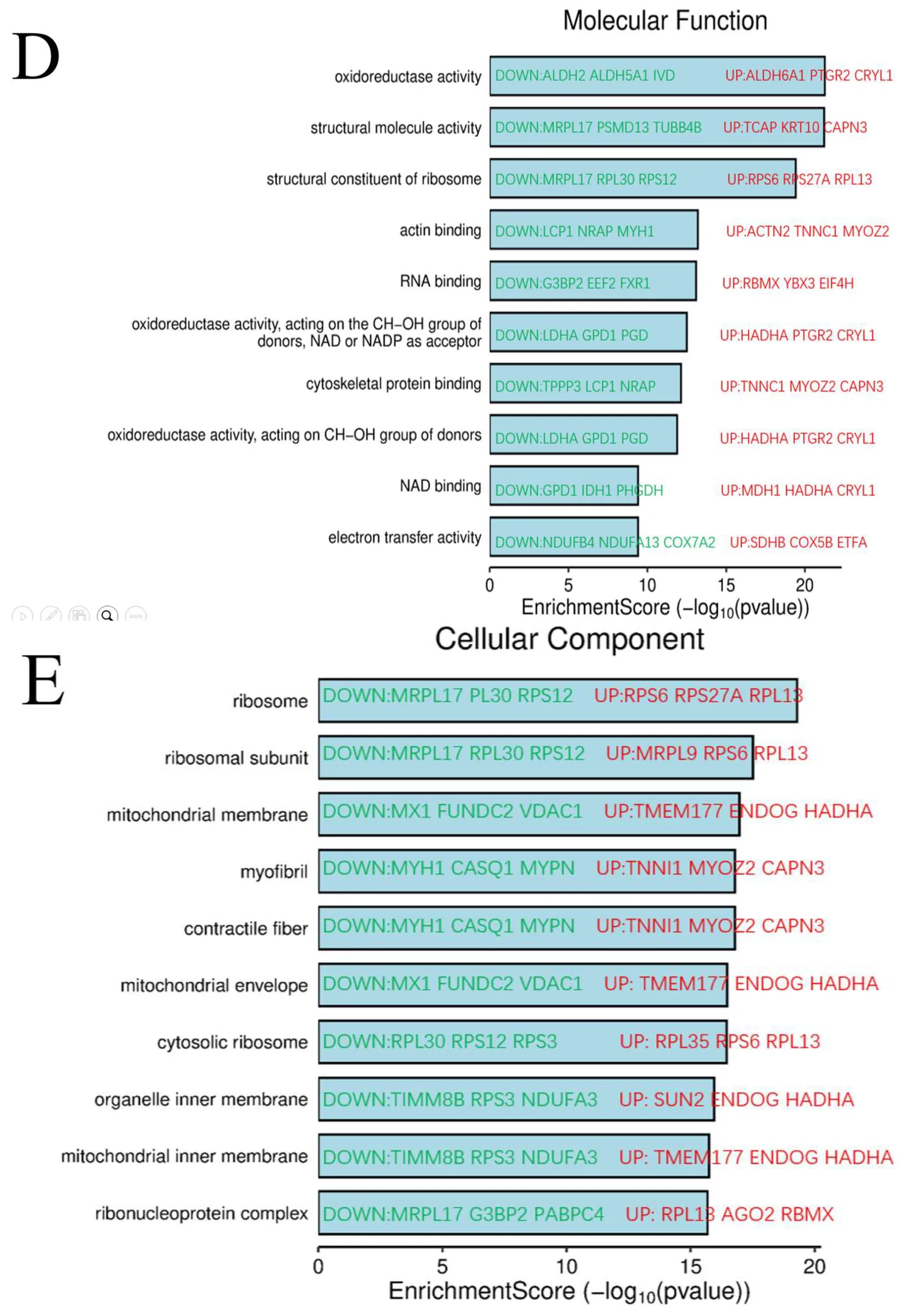

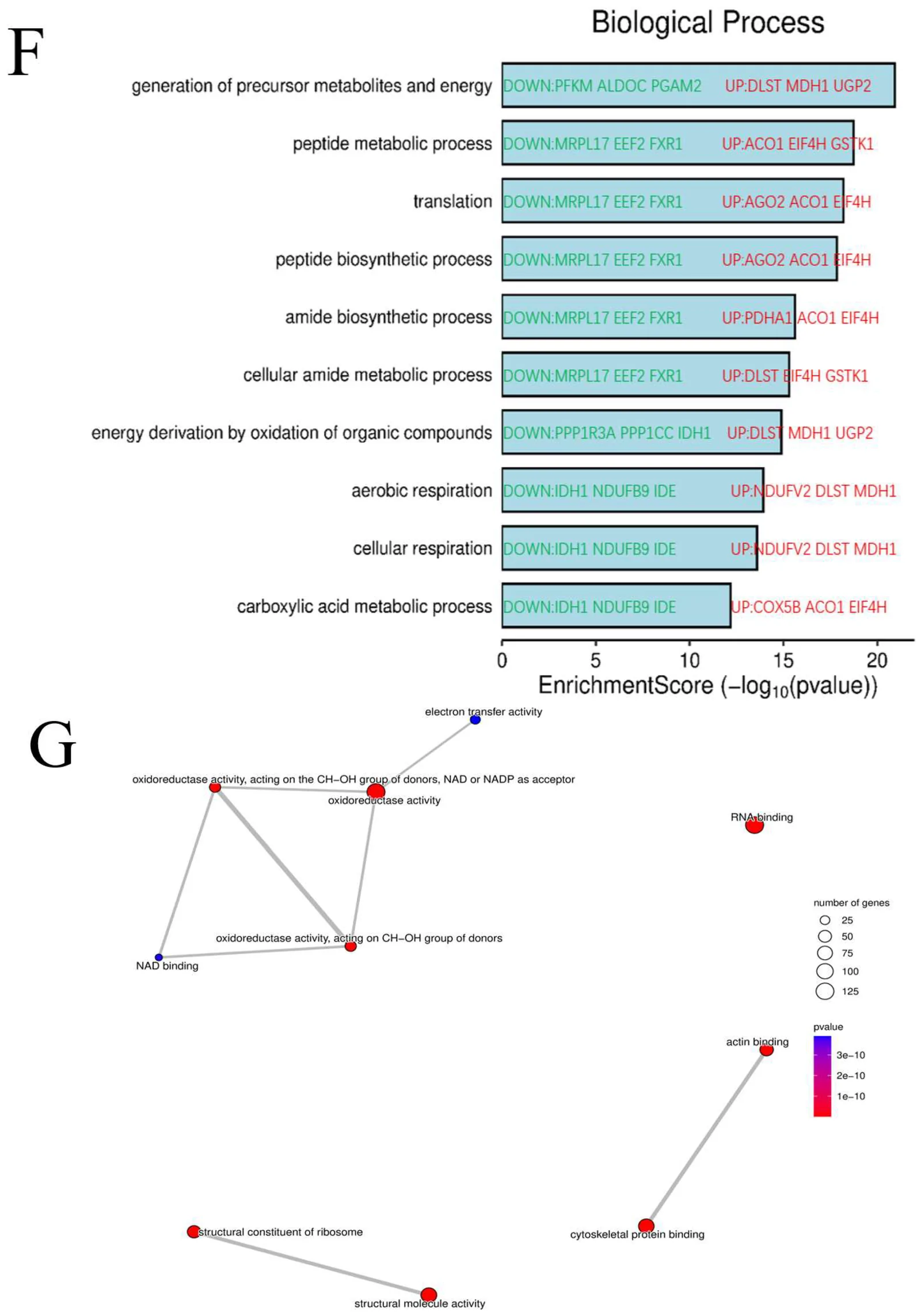

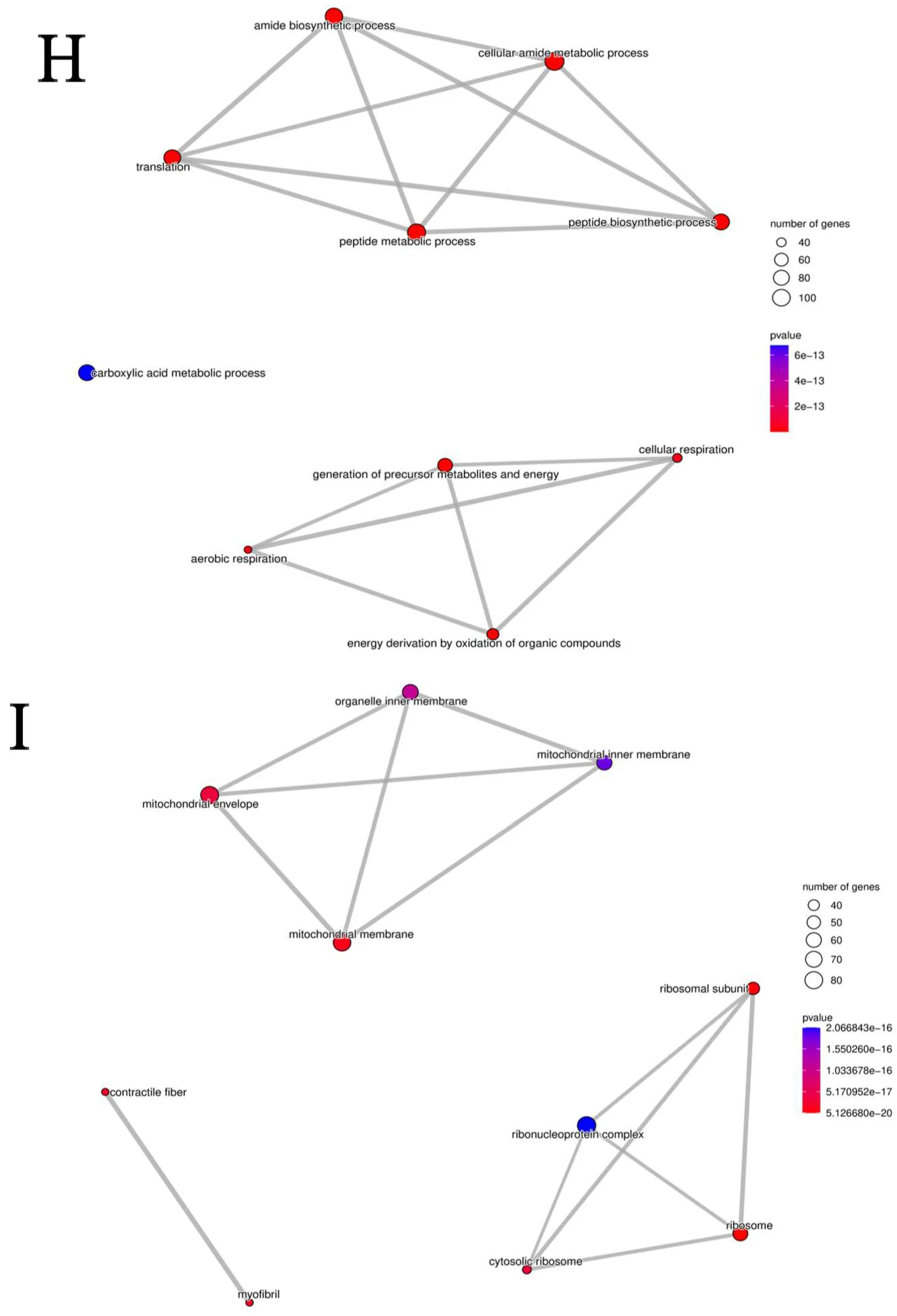
Results Figure of GO Clustering Analysis of Differential Proteins between Large White Pigs and Bama Pigs. (**A**) Schematic Diagram of Skeletal Muscle Tissue of Large White Pigs and Bama Pigs. (**B**) Volcano Plot of Differential Proteins in the Longissimus Dorsi Muscle Between Large White Pigs and Bama Xiang Pigs. (**C**) Protein Expression Clustering Heatmap of Large White Pigs and Bama Pigs. (**D**) Bar Chart of Molecular Function Clustering Analysis and Significantly Differential Proteins of Large White Pigs and Bama Pigs. (**E**) Bar Chart of Biological Process Clustering Analysis and Significantly Differential Proteins of Large White Pigs and Bama Pigs. (**F**) Bar Chart of Cellular Component Clustering Analysis and Significantly Differential Proteins of Large White Pigs and Bama Pigs. (**G**) Network Diagram of Molecular Function Clustering Analysis of Large White Pigs and Bama Pigs. (**H**) Network Diagram of Biological Process Clustering Analysis of Large White Pigs and Bama Pigs. (**I**) Network Diagram of Cellular Component Clustering Analysis of Large White Pigs and Bama Pigs.

ITRAQ-based proteomic analysis of longissimus dorsi muscle tissues from Large White pigs and Bama pigs identified a total of 2040 reliably expressed proteins. Compared with Bama pigs, 51 proteins were upregulated and 73 proteins were downregulated in Large White pigs. Proteins exhibiting a 1.3-fold change as the threshold for differential expression are shown in Figure 2B. The differentially expressed proteins were mainly enriched in pathways related to energy metabolism, muscle fiber structural and functional regulation, and protein synthesis (Figure 2G–I). Among these pathways, glycolysis is a key metabolic route that directly influences skeletal muscle growth and metabolism. Within the glycolytic pathway, the expression levels of PFKM, ALDH2 (Aldehyde Dehydrogenase 2), and LDHA (Lactate Dehydrogenase A) were significantly increased in the comparison of Large White pigs versus Bama pigs, whereas the expression levels of GPI (Glucose-6-phosphate Isomerase), ACSS2 (Acyl-CoA Synthetase Short-chain Family Member 2), and LDHB (Lactate Dehydrogenase B) were significantly decreased in the comparison of Large White pigs versus Bama pigs (Figure 2D–F). Based on its central role in glycolysis, PFKM was selected as a key candidate protein for subsequent investigations.

### 3.2. Effects of Altered PFKM Expression on Apoptosis in SMSCs

**Figure 3:**
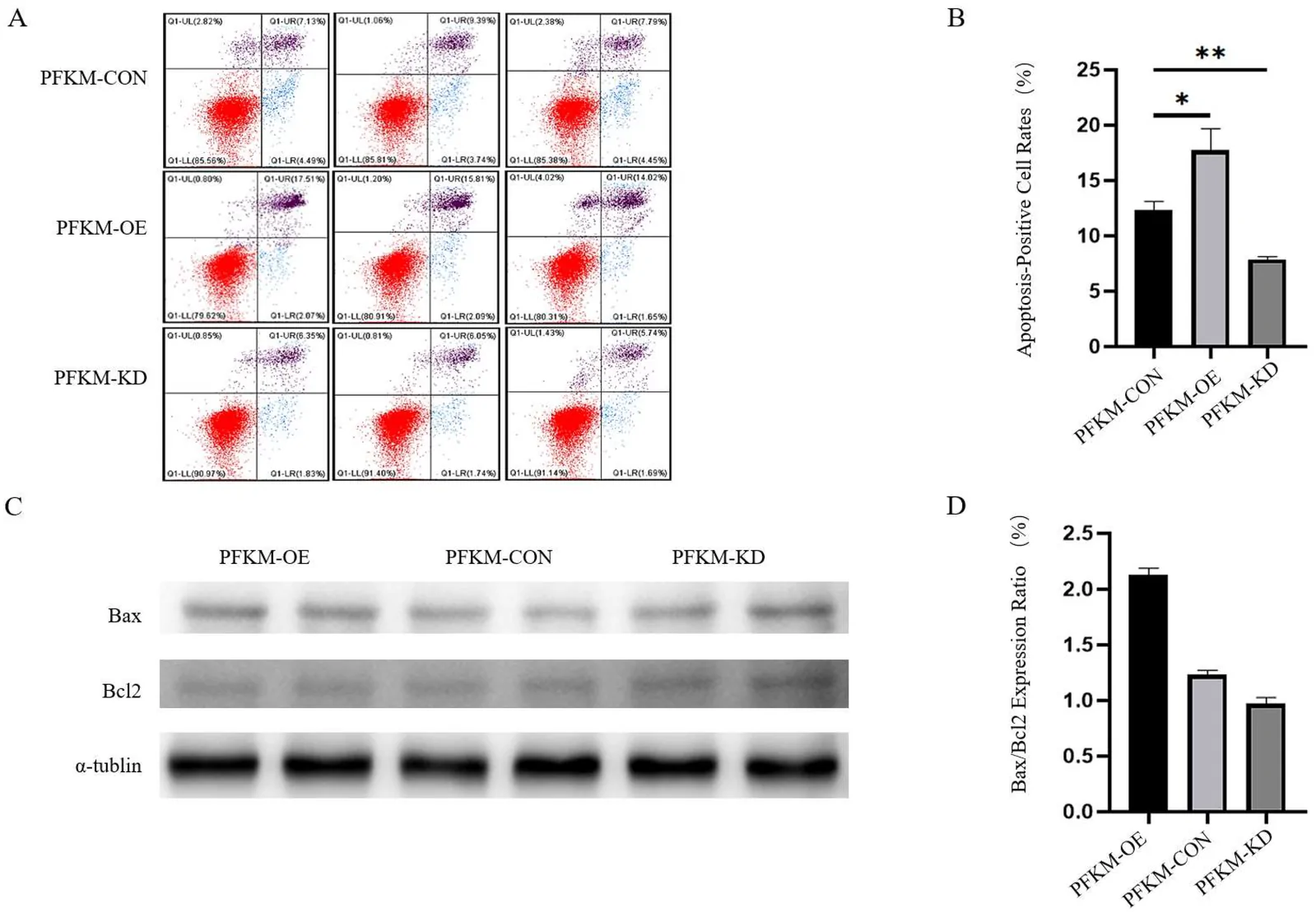
Detection of cell apoptosis level. (**A**) Detection of cell apoptosis level by flow cytometry. (**B**) Analysis of the results of cell apoptosis detection. (**C**) Detection of apoptosis levels in three cell groups by Western blot. (**D**) Analysis of the results of cell apoptosis detection by Western blot. * *p* < 0.05. ** *p* < 0.01.

The apoptosis assay results for each group are shown in Figure 3. The normal control group was designated as PFKM-CON, the PFKM gene knockdown group as PFKM-KD, and the PFKM overexpression group as PFKM-OE. Flow Cytometry analysis was performed to detect the expression of the apoptosis-related proteins Bcl-2 and Bax. In the Annexin V/PI flow cytometric analysis, cells in the lower-left quadrant, shown in red, were Annexin V-negative and PI-negative and were therefore classified as viable cells. Cells in the lower-right quadrant, shown in blue, were Annexin V-positive and PI-negative, representing early apoptotic cells. Cells in the upper-right quadrant were positive for both Annexin V and PI and were classified as late apoptotic or secondarily necrotic cells. Cells in the upper-left quadrant were Annexin V-negative and PI-positive, generally representing primarily necrotic or membrane-damaged cells. The total apoptotic rate was calculated as the sum of the early and late apoptotic populations, corresponding to the lower-right and upper-right quadrants, respectively.The apoptosis rate in the control group was 12.4% ± 0.4%. Compared with the control group, the apoptosis rate in the PFKM overexpression group was significantly increased (*p* < 0.05), reaching 17.7% ± 0.7%, whereas the apoptosis rate in the PFKM knockdown group was slightly decreased, to approximately 7.1% ± 0.2% (Figure 3A,B). Based on Western blot analysis, the Bax/Bcl-2 relative expression ratios in the three groups were 2.12 ± 0.08, 1.27 ± 0.07, and 0.94 ± 0.08, (Due to limitations in experimental progress and scheduling at the time, only two valid independent Western blot replicates were obtained for Bax and Bcl-2. Therefore, these results were used only to support the flow cytometry findings and to describe the overall trend in apoptosis. Their statistical reliability is limited, and no inferential statistical analysis was performed.), showing an overall trend consistent with the results (Figure 3C,D). These findings indicate that PFKM overexpression significantly increases apoptosis in SMSCs, whereas PFKM knockdown reduces the apoptotic rate.

### 3.3. Effects of Altered PFKM Expression on Mitochondrial Membrane Potential in SMSCs as Assessed by Flow Cytometry

**Figure 4:**
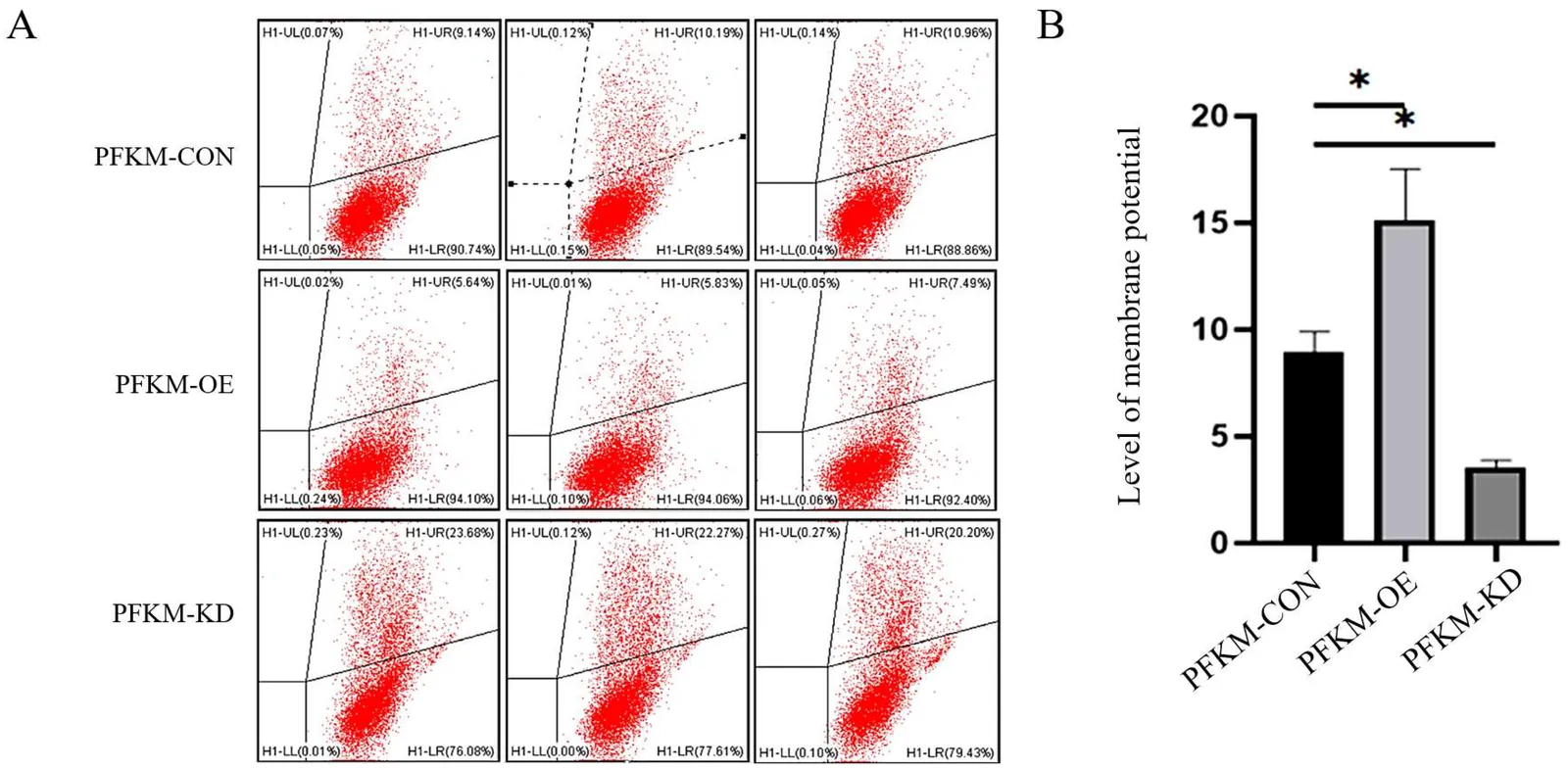
Detection of the mitochondrial membrane potential levels of cells. (**A**) Detection of the mitochondrial membrane potential level of cells by flow cytometry. (**B**) Analysis of the detection results of the mitochondrial membrane potential of cells. * *p* < 0.05.

The flow cytometric analysis of mitochondrial membrane potential in each group is shown in Figure 4. Cells were divided into three groups: the normal control group (PFKM-CON), the PFKM gene knockdown group (PFKM-KD), and the PFKM overexpression group (PFKM-OE). The JC-1(5,5′,6,6′-tetrachloro-1,1′,3,3′-tetraethylbenzimidazolocarbocyanine iodide) red/green fluorescence ratio in the control group was 8.9 ± 0.9. Compared with the control group, the JC-1 red/green ratio was significantly decreased in the PFKM knockdown group (*p* < 0.05), to 3.6 ± 0.3, whereas it was significantly increased in the PFKM overexpression group (*p* < 0.05), to 15.0 ± 2.4 (Figure 4A,B). These results indicate that PFKM knockdown reduces mitochondrial membrane potential in SMSCs, whereas PFKM overexpression elevates mitochondrial membrane potential.

### 3.4. Effects of Altered PFKM Expression on Reactive Oxygen Species (ROS) Levels in SMSCs as Assessed by Flow Cytometry

**Figure 5:**
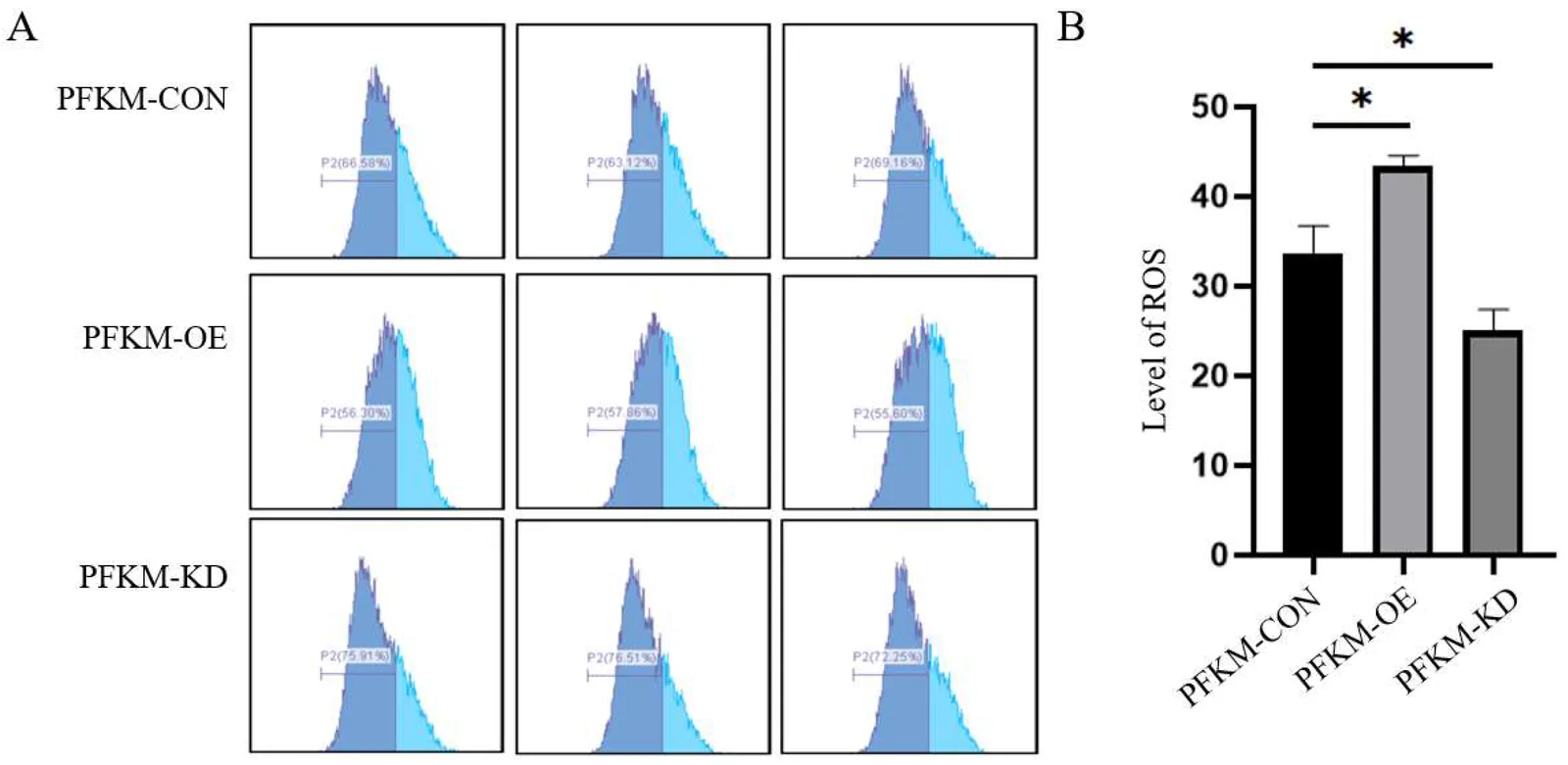
Detection of the levels of reactive oxygen species. (**A**) Detection of the level of mitochondrial reactive oxygen species (ROS) in cells by flow cytometry. (**B**) Analysis of the detection results of the level of mitochondrial reactive oxygen species (ROS) in cells. * *p* < 0.05.

The flow cytometric analysis of intracellular reactive oxygen species (ROS) levels in each group is shown in Figure 5. Cells were divided into three groups: the normal control group (PFKM-CON), the PFKM gene knockdown group (PFKM-KD), and the PFKM overexpression group (PFKM-OE). P2 represents a fixed analysis gate with the same position and range applied to all samples. Events falling within the P2 gate are displayed in dark blue. A larger dark-blue proportion indicates that more cells are distributed within the low-fluorescence region, suggesting a lower overall intracellular ROS level. Conversely, a larger light-blue proportion indicates that more cells are distributed in the high-fluorescence region, reflecting an overall increase in ROS-associated fluorescence and therefore a higher ROS level in the cell population.The ROS level in the control group was 33.7 ± 3.1. Compared with the control group, the ROS level in the PFKM knockdown group was significantly reduced (*p* < 0.05), to 25.1 ± 2.3, whereas the ROS level in the PFKM overexpression group was significantly increased (*p* < 0.05), to 43.4 ± 1.2 (Figure 5A,B). These results indicate that PFKM knockdown decreases ROS production in SMSCs, whereas PFKM overexpression elevates intracellular ROS levels.

### 3.5. Effects of Altered PFKM Expression on ATP Levels in SMSCs as Measured by a Microplate Reader

**Figure 6:**
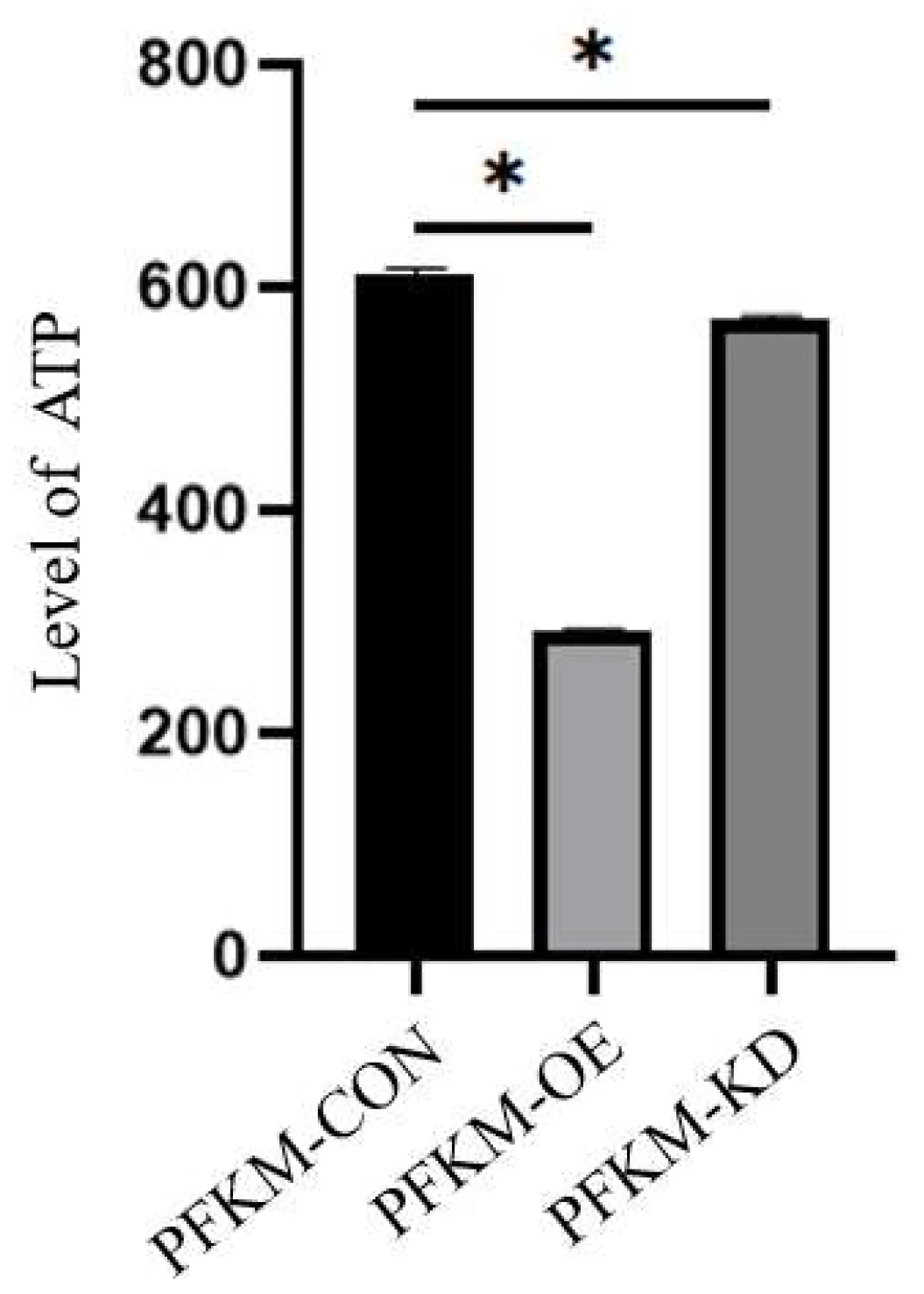
Detection of ATP levels. * *p* < 0.05.

The ATP assay results for each group are shown in Figure 6. Cells were divided into three groups: the normal control group (PFKM-CON), the PFKM gene knockdown group (PFKM-KD), and the PFKM overexpression group (PFKM-OE). After large-scale culture, ATP levels in each group were measured using an ATP assay kit and a fluorescence microplate reader. The relative ATP level in the control group was 612 ± 15. Compared with the control group, the relative ATP level in the PFKM knockdown group was significantly reduced (*p* < 0.05), to approximately 573 ± 9, and it was markedly decreased in the PFKM overexpression group (*p* < 0.05), to 291 ± 8. These results indicate that both PFKM knockdown and PFKM overexpression lead to a reduction in mitochondrial ATP content in SMSCs.

### 3.6. Effects of Altered PFKM Expression on the Expression of Mitochondrial Function–Related Proteins in SMSCs as Determined by Western Blot

**Figure 7:**
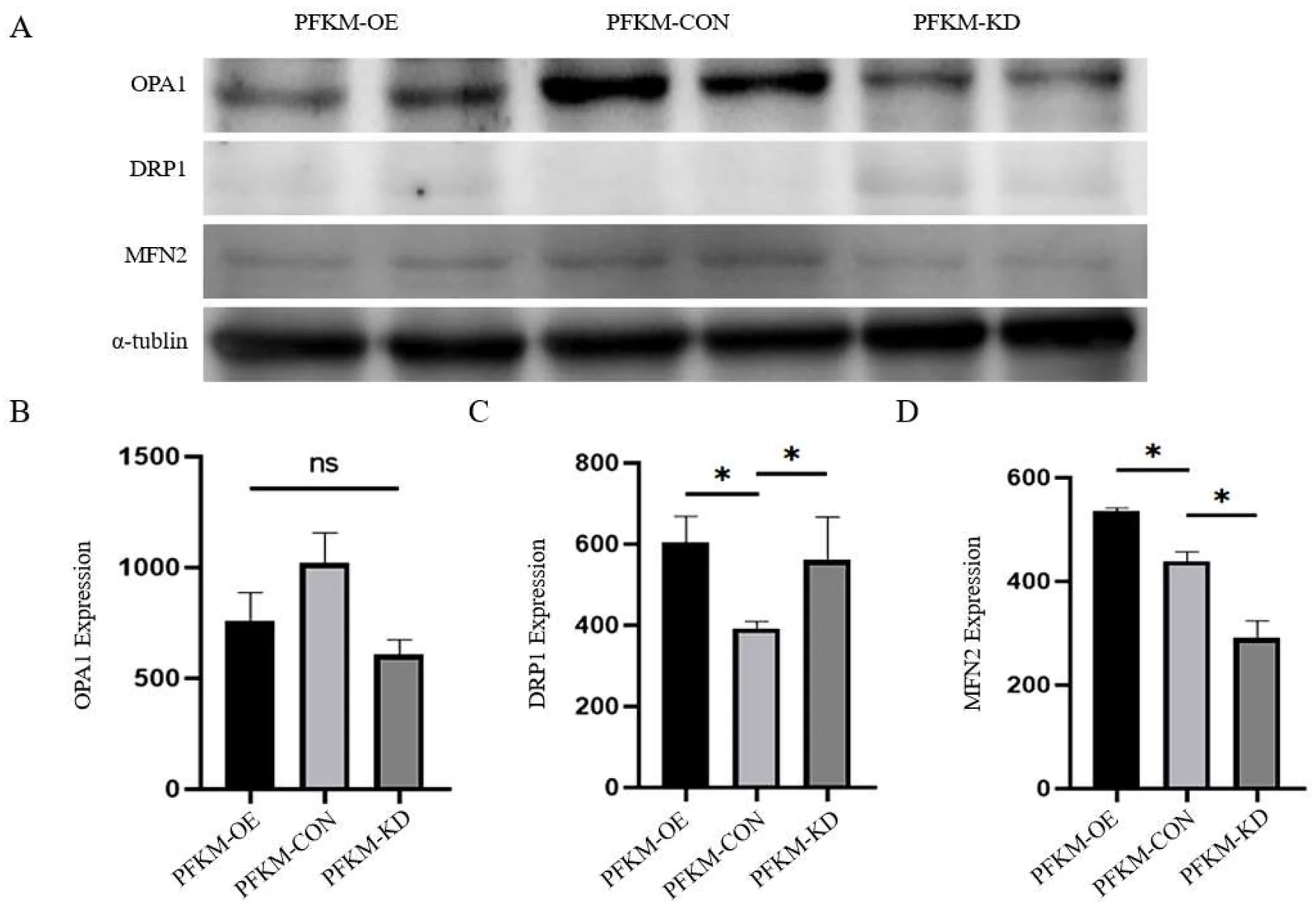
Detection of the protein levels of mitochondrial function. (**A**) Schematic diagram of the protein gray-scale expression. (**B**) Analysis diagram of the gray-scale expression of OPA1 protein. (**C**) Analysis diagram of the gray-scale expression of DRP1 protein. (**D**) Analysis diagram of the gray-scale expression of MFN2 protein. * *p* < 0.05.

The expression levels of mitochondrial function–related proteins in each group are shown in Figure 7. Cells were divided into three groups: the normal control group (PFKM-CON), the PFKM gene knockdown group (PFKM-KD), and the PFKM overexpression group (PFKM-OE). Total cellular protein was extracted from each group, with α-tubulin used as the internal control. Western blotting was performed to detect the expression of mitochondrial function–related proteins, and band intensities were quantified by grayscale analysis. Based on Western blot analysis, the relative expression levels of OPA1 in the three groups were 763 ± 148, 1017 ± 169, and 589 ± 81, respectively (Owing to limitations in the batch stability, detection efficiency, and available stock of the OPA1 antibody stored in our laboratory, only two independent Western blot replicates with clearly confirmed antibody specificity were obtained for OPA1. Accordingly, the OPA1 results were considered only as preliminary evidence of changes related to mitochondrial dynamics. Their statistical reliability is limited, and no inferential statistical analysis was performed.) (Figure 7B). The relative expression levels of DRP1 were 603 ± 89, 391 ± 22, and 513 ± 126, respectively (*p* < 0.05) (Figure 7C). The relative expression levels of MFN2 were 546 ± 14, 433 ± 30, and 372 ± 51, respectively (*p* < 0.05) (Figure 7D), with the CON group used as the reference control. Compared with the CON group, OPA1 protein expression was decreased in both the PFKM-OE and PFKM-KD groups. DRP1 protein expression was significantly increased in both the PFKM-OE and PFKM-KD groups compared with the CON group (*p* < 0.05). In contrast, MFN2 protein expression was significantly increased in the PFKM-OE group (*p* < 0.05) but significantly decreased in the PFKM-KD group (*p* < 0.05) relative to the CON group.

### 3.7. Effects of Altered PFKM Expression on Myogenic Differentiation of SMSCs

#### 3.7.1. Western Blot Analysis of the Effects of Altered PFKM Expression on Myogenic Differentiation

**Figure 8:**
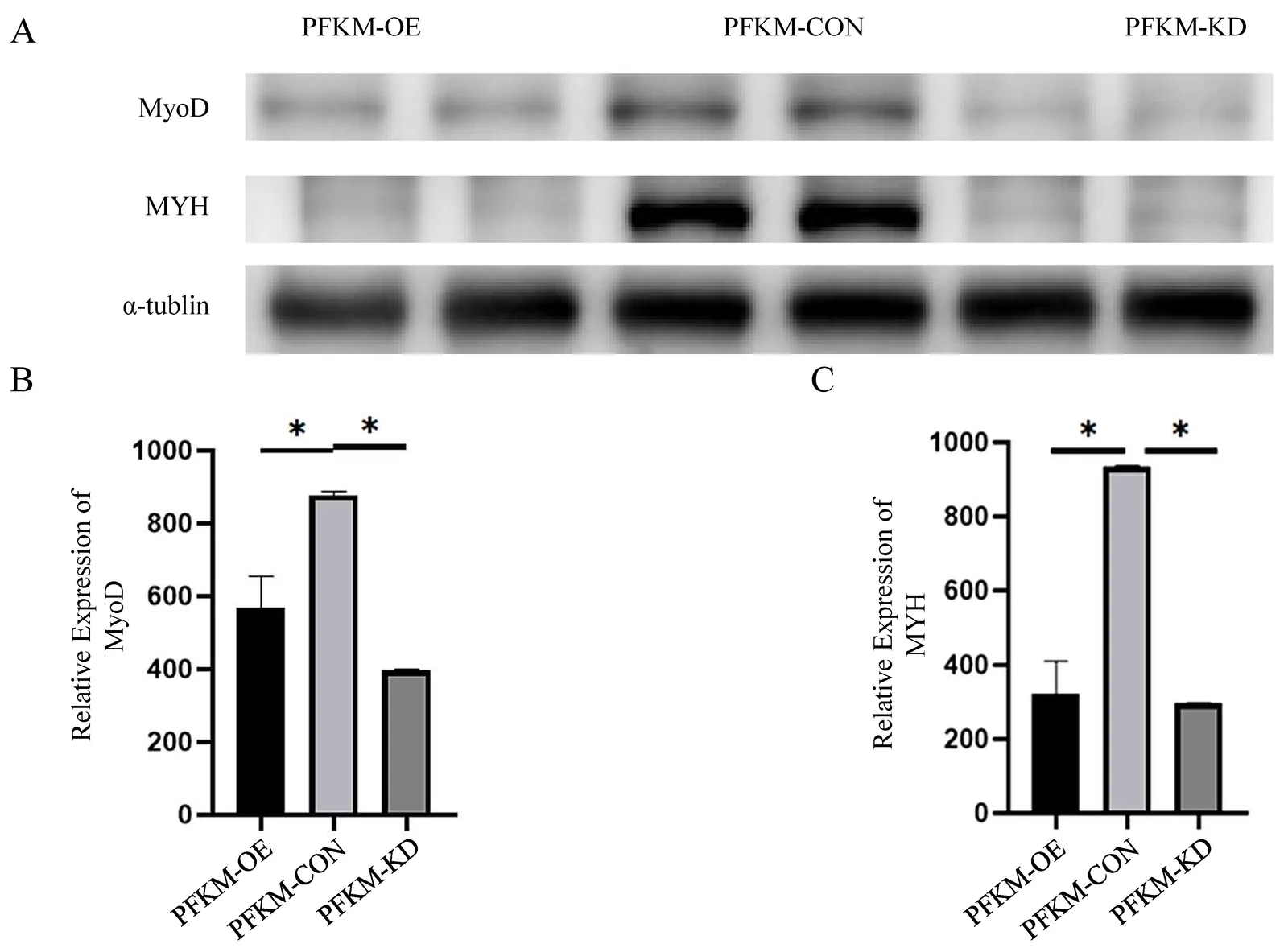
Detection of the protein levels of mitochondrial function. (**A**) Diagram of the gray-scale expression of MYoD protein. (**B**) Analysis diagram of the gray-scale expression of MYoD protein. (**C**) Analysis diagram of the gray-scale expression of MYH protein. * *p* < 0.05.

The expression levels of myogenic differentiation–related proteins in each group are shown in Figure 8. Cells were divided into three groups: the normal control group (PFKM-CON), the PFKM gene knockdown group (PFKM-KD), and the PFKM overexpression group (PFKM-OE). Total cellular protein was extracted from each group, with α-tubulin used as the internal control. Western blot analysis was performed to detect the expression of MyoD and MYH proteins, and band intensities were quantified by grayscale analysis. The relative expression levels of MyoD in the three groups were 557 ± 102, 873 ± 15, and 391 ± 8, respectively (*p* < 0.05) (Figure 8B). The relative expression levels of MYH were 317 ± 103, 922 ± 6, and 387 ± 4, respectively (*p* < 0.05) (Figure 8C). Compared with the control group, both the PFKM knockdown and PFKM overexpression groups showed significantly reduced expression of MyoD and MYH.

#### 3.7.2. Immunofluorescence Analysis of the Effects of Altered PFKM Expression on Myogenic Differentiation of SMSCs

**Figure 9:**
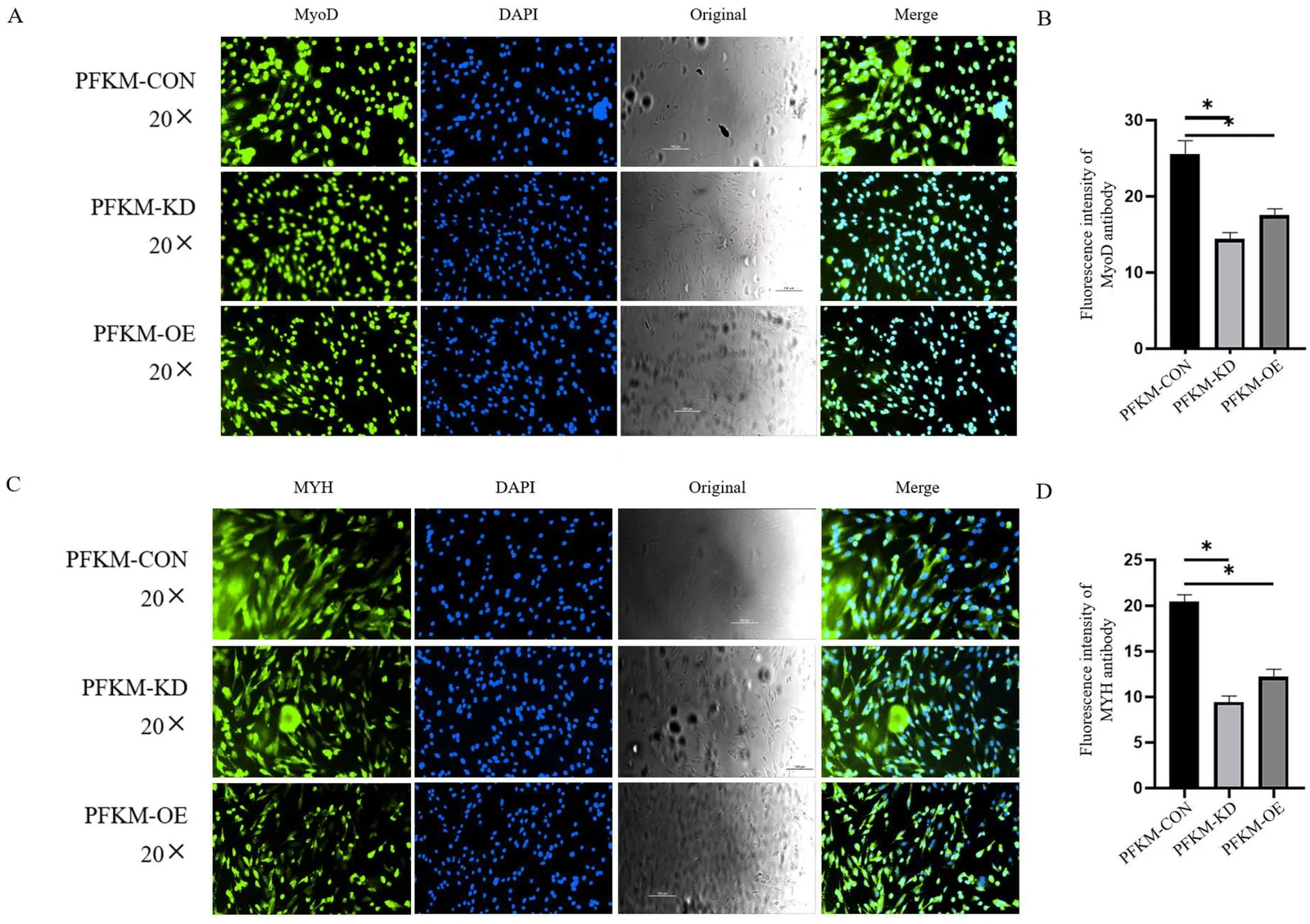
Detection of the protein levels of mitochondrial function. (**A**) Schematic diagram of the immunofluorescence intensity of MYoD. (**B**) Analysis diagram of the immunofluorescence intensity of MYoD. (**C**) Schematic diagram of the immunofluorescence intensity of MYH. (**D**) Analysis diagram of the immunofluorescence intensity of MYH. * *p* < 0.05.

The immunofluorescence results for myogenic differentiation–related protein expression in each group are shown in Figure 9. Cells were divided into three groups: the normal control group (PFKM-CON), the PFKM gene knockdown group (PFKM-KD), and the PFKM overexpression group (PFKM-OE). After 96 h of culture in myogenic differentiation induction medium, fluorescence intensity was examined by immunofluorescence staining. The relative fluorescence intensities of MyoD in the three groups were 25.08 ± 2.24, 14.00 ± 0.93, and 17.00 ± 0.92, respectively (*p* < 0.05) (Figure 9B). The relative fluorescence intensities of MYH were 20.27 ± 0.91, 9.32 ± 0.89, and 11.80 ± 0.98, respectively (*p* < 0.05) (Figure 9D). The expression levels of MyoD and MYH in the control group were significantly higher than those in both the PFKM knockdown and PFKM overexpression groups (*p* < 0.05), which is consistent with the Western blot results.

### 3.8. Effects of Altered PFKM Expression on Glycolytic Activity in SMSCs

**Figure 10:**
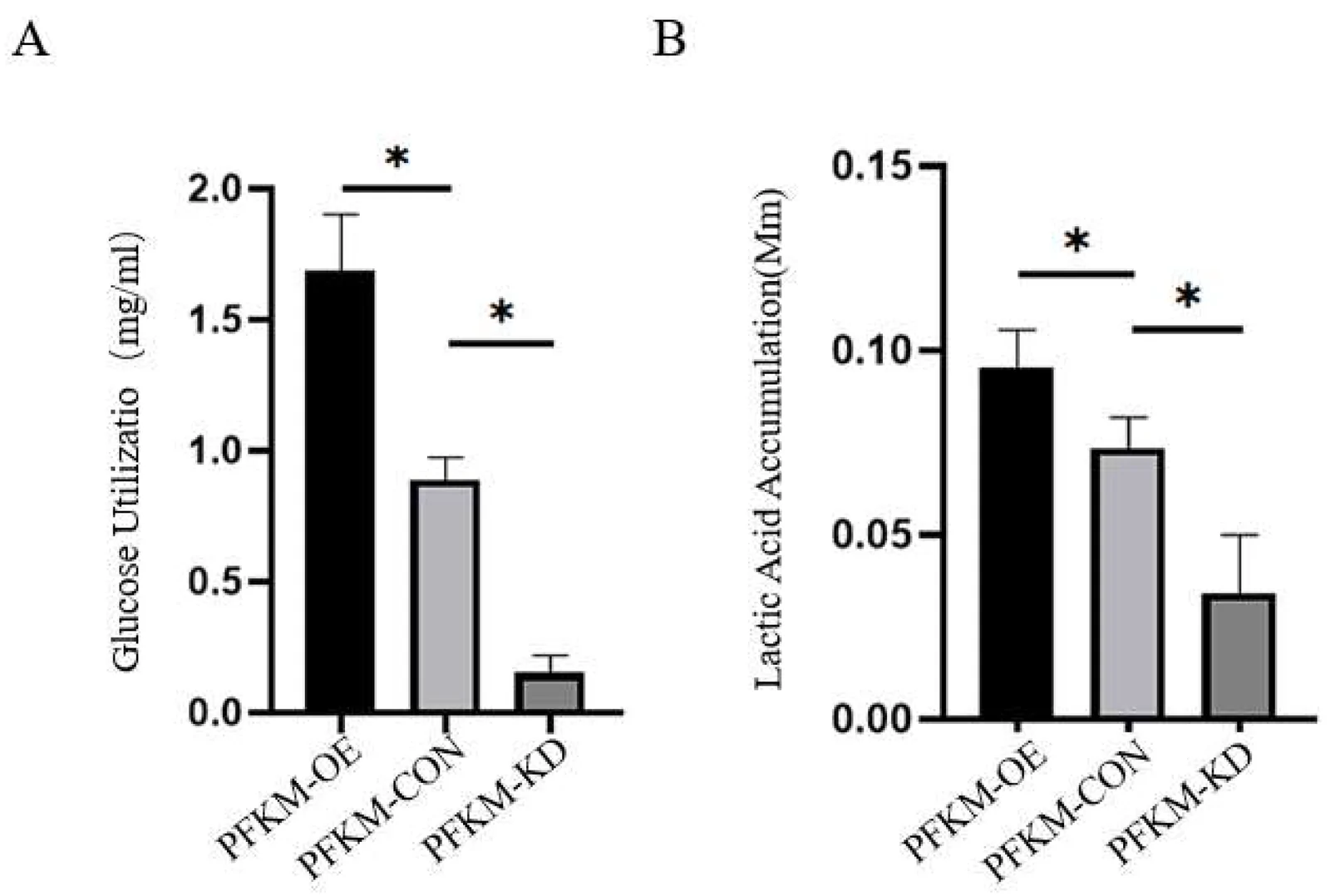
Detection results of glycolysis level in PFKM overexpression and knockout cell populations. (**A**) Schematic diagram of the detection of cellular glucose utilization rate. (**B**) Schematic diagram of the detection of cellular glucose utilization rate. * *p* < 0.05.

The results of glycolytic activity analysis in each group are shown in Figure 10. Cells were divided into three groups: the normal control group (PFKM-CON), the PFKM gene knockdown group (PFKM-KD), and the PFKM overexpression group (PFKM-OE). Differences in glucose and lactate concentrations were calculated relative to fresh culture medium. The glucose uptake levels in the three groups were 1.67 ± 0.18, 0.87 ± 0.01, and 0.14 ± 0.01, respectively (*p* < 0.05) (Figure 10A), while the lactate accumulation levels were 0.09 ± 0.01, 0.07 ± 0.01, and 0.03 ± 0.02, respectively (*p* < 0.05) (Figure 10B). These results indicate that the PFKM overexpression group exhibited significantly higher glucose consumption and lactate accumulation than the control group (*p* < 0.05), reflecting significantly enhanced glycolytic activity. In contrast, the PFKM knockdown group showed significantly lower glucose consumption and lactate accumulation compared with the control group (*p* < 0.05), indicating a marked reduction in glycolytic activity.

## 4. Discussion

In this study, Large White pigs and Bama miniature pigs, which differ markedly in growth rate, body size, and skeletal muscle metabolic characteristics, were used as comparative models. Differentially expressed proteins in skeletal muscle were first screened using iTRAQ-based proteomics. Subsequently, PFKM, a key rate-limiting enzyme in glycolysis, was selected as a candidate gene, and its function was investigated in porcine skeletal muscle satellite cells through overexpression and knockdown experiments. The results demonstrated that skeletal muscle from the two pig breeds differed in glycolysis, mitochondrial energy metabolism, muscle fiber structure, and protein synthesis. Further cellular experiments showed that dysregulated PFKM expression altered glucose utilization, lactate production, ATP levels, redox status, and mitochondrial function-related parameters, accompanied by reduced expression of the myogenic markers MyoD and MYH. These findings preliminarily support the scientific hypothesis proposed in this study that PFKM may be an important candidate regulator linking glycolytic metabolism, cellular energy homeostasis, and myogenic differentiation in skeletal muscle. However, the present study demonstrated associations between altered PFKM expression and the observed phenotypes, rather than establishing a complete and reliable causal mechanism involving downstream signaling pathways such as the AMPK–DRP1 axis.

Proteomic analysis identified a total of 2040 reliably expressed proteins. Among them, 51 proteins were upregulated in Large White pigs relative to Bama miniature pigs, whereas 73 proteins were upregulated in Bama miniature pigs relative to Large White pigs. These differentially expressed proteins were mainly enriched in processes related to protein synthesis, glycolysis, the tricarboxylic acid cycle, oxidative phosphorylation, and the regulation of muscle fiber structure and function. Based on the expression patterns of PFKM and other metabolism-related proteins, the results suggest that skeletal muscle from Large White pigs may rely more heavily on rapid glycolytic energy production, whereas skeletal muscle from Bama miniature pigs may possess a relatively greater capacity for mitochondrial oxidative metabolism. Previous proteomic studies of porcine skeletal muscle have similarly demonstrated marked differences in the expression of glycolytic enzymes, tricarboxylic acid cycle enzymes, and respiratory-chain proteins among animals with different genetic backgrounds and muscle fiber compositions. These differences may be associated with growth rate, muscle fiber composition, and meat quality traits [18,19,20]. Therefore, the breed-related differences in metabolic proteins observed in the present study are biologically plausible. Nevertheless, owing to the limited sample size, these findings should be interpreted as preliminary trends in skeletal muscle metabolic patterns between the two pig breeds rather than as definitive breed-wide metabolic characteristics.

PFKM catalyzes the conversion of fructose-6-phosphate to fructose-1,6-bisphosphate and occupies a major regulatory position in glycolysis. Changes in its expression or activity may therefore simultaneously affect energy production, the distribution of metabolic intermediates, and redox balance. In the present study, glucose consumption and lactate accumulation changed in parallel, suggesting that PFKM knockdown reduced glycolysis-related substrate utilization in SMSCs, whereas PFKM overexpression enhanced glucose utilization and lactate production. Recent studies have shown that PFKM is maintained at a relatively low level in muscle stem cells and gradually increases during differentiation. PFKM knockdown has been reported to reduce both glycolysis and oxidative phosphorylation and to inhibit differentiation, whereas supplementation with downstream glycolytic intermediates can partially restore the differentiation phenotype. These findings further support the involvement of PFKM and glycolytic intermediates in regulating muscle cell fate [21,22].

Previous studies have demonstrated a positive association between PFKM expression and glycolytic activity [23,24]. However, it should be noted that although the PFKM-OE group exhibited increased glucose consumption and lactate accumulation, its relative ATP level was significantly reduced. This result indicates that increased substrate consumption was not translated into effective cellular energy accumulation. This phenomenon may reflect a mismatch among glycolytic flux, pyruvate oxidation, mitochondrial ATP synthesis, and cellular energy expenditure. The increased metabolic burden caused by PFKM overexpression may direct a greater proportion of glucose-derived carbon toward lactate production or increase ATP consumption required to maintain ion homeostasis, antioxidant defenses, protein repair, and apoptotic processes [25]. In comparison, the reduction in ATP levels was considerably less pronounced in the PFKM-KD group than in the overexpression group and was accompanied by marked reductions in glucose consumption and lactate accumulation. This pattern is more consistent with an overall restriction of cellular metabolic activity [26]. Therefore, PFKM overexpression may primarily induce oxidative stress, ATP depletion, and increased apoptosis, whereas PFKM knockdown may mainly reduce mitochondrial membrane potential, ROS production, and ATP levels, resulting in a relatively hypometabolic state. Both treatments caused the cells to deviate from normal metabolic homeostasis, but the directions of the changes and the nature of the resulting cellular impairments were not identical.

Measurements related to mitochondrial function further demonstrated these differences. Compared with the control group, the PFKM-OE group showed an increased JC-1 red-to-green fluorescence ratio, elevated ROS levels, and an increased apoptotic rate. The simultaneous occurrence of increased mitochondrial membrane potential and markedly decreased ATP levels indicates that a higher JC-1 ratio cannot simply be interpreted as enhanced mitochondrial energy-producing capacity. Instead, it may reflect mitochondrial hyperpolarization, impaired coordination between the respiratory chain and ATP synthesis or utilization, or abnormal maintenance of the proton gradient under cellular stress [27]. In contrast, the PFKM-KD group exhibited a lower JC-1 ratio, reduced ROS levels, and a lower apoptotic rate than the control group. These findings indicate that PFKM knockdown did not produce the same oxidative damage phenotype as PFKM overexpression. Rather, reduced glycolytic substrate supply and lower overall metabolic activity may have caused mitochondrial membrane potential, ROS signaling, and ATP production to decline simultaneously [28].

ROS have dual roles during myogenesis. Moderate levels of ROS can act as signaling molecules involved in cellular proliferation, metabolic adaptation, and differentiation. However, ROS accumulation beyond the buffering capacity of the antioxidant system can cause oxidative damage to proteins, lipids, and nucleic acids, thereby impairing muscle cell function and potentially inducing apoptosis. Conversely, excessive suppression of ROS and the development of an overly reducing intracellular environment may weaken the redox signals required for normal differentiation [29]. In the present study, the increase in ROS in the PFKM-OE group was accompanied by ATP depletion and increased apoptosis, suggesting that the ROS elevation in this group was more likely associated with pathological oxidative stress. In the PFKM-KD group, reduced ROS levels and a lower apoptotic rate may reflect reduced metabolic activity, but they may also indicate insufficient physiological ROS signaling required for myogenic differentiation. These results suggest that normal differentiation of SMSCs may require PFKM expression, energy supply, and ROS signaling to remain within an appropriate range rather than simply requiring either higher or lower glycolytic activity.

Changes in mitochondrial dynamics-related proteins also suggested that dysregulated PFKM expression was accompanied by abnormal regulation of mitochondrial homeostasis. Compared with the PFKM-CON group, OPA1 expression was reduced and DRP1 expression was increased in both the PFKM-OE and PFKM-KD groups. However, MFN2 expression was increased in the PFKM-OE group and decreased in the PFKM-KD group. The simultaneous reduction in OPA1 and MFN2 and increase in DRP1 in the PFKM-KD group may indicate weakened support for mitochondrial fusion together with enhanced regulation associated with mitochondrial fission. In the PFKM-OE group, the concurrent increases in DRP1 and MFN2 may represent simultaneous disturbance of both fission and fusion regulation or a compensatory response to metabolic stress. Previous studies have shown that DRP1-dependent mitochondrial remodeling and mitophagy participate in normal myogenic differentiation, and that excessive inhibition or disruption of mitochondrial fission can impair mitochondrial renewal, myotube formation, and myogenic gene expression [30]. Therefore, mitochondrial dynamics cannot be interpreted simply as “fission is harmful and fusion is beneficial”; instead, a dynamic balance must be maintained at specific stages of differentiation. The present results support an association between abnormal PFKM expression and imbalances in mitochondrial dynamics-related proteins, but they are insufficient to demonstrate that mitochondrial network fragmentation occurred.

The myogenic differentiation results constitute the central functional validation of this study. MYH and MyoD are key myogenic markers [31]. Western blot analysis showed that the relative expression levels of both MyoD and MYH were reduced in the PFKM-OE group compared with the PFKM-CON group. The relative expression levels of MyoD and MYH were also reduced in the PFKM-KD group. Immunofluorescence analysis performed after 96 h of myogenic induction produced consistent results. These findings indicate that either excessive or insufficient PFKM expression is associated with reduced myogenic differentiation capacity in SMSCs, supporting the possibility that a nonlinear optimal range exists between PFKM expression and myogenic function [32].

Notably, a recent study using human muscle-derived cells found that PFKM knockdown inhibited differentiation, whereas PFKM overexpression under certain conditions increased the expression of differentiation markers such as MyHC [33]. This finding is not entirely consistent with the reductions in MyoD and MYH observed in the PFKM-OE group in the present study. The discrepancy may be related to differences in species, cell origin, stage of differentiation, magnitude of overexpression, and duration of PFKM expression. A moderate and appropriately timed increase in PFKM expression may provide glycolytic intermediates and energy to support differentiation, whereas sustained or excessive PFKM expression may cause a mismatch between glycolysis and mitochondrial oxidative capacity. The PFKM-OE group in the present study simultaneously exhibited increased ROS levels, severe ATP depletion, and increased apoptosis, suggesting that its metabolic state had exceeded the range favorable for differentiation. Therefore, the present findings do not negate the requirement for PFKM during normal myogenic differentiation but instead suggest that its effects may be dependent on both dose and timing.

This study has several limitations. First, the proteomic analysis included only three biological samples from each pig breed. Although this sample size is suitable for exploratory screening, its statistical power is limited, and individual genetic background and physiological status may influence the identification of differentially expressed proteins. Therefore, breed-related metabolic differences and the proposed candidate role of PFKM require validation in larger independent populations. Future studies should also incorporate sex, age, rearing conditions, muscle fiber type, and meat quality phenotypes into the analysis.

Second, glucose consumption and lactate accumulation in the culture medium were used as glycolysis-related indicators, but these measurements cannot directly represent real-time glycolytic flux. Future studies should use extracellular flux analysis to measure the extracellular acidification rate and oxygen consumption rate, allowing evaluation of basal glycolysis, glycolytic capacity, glycolytic reserve, basal respiration, maximal respiration, and ATP-linked respiration. Stable isotope-labeled glucose tracing should also be used to determine the distribution of glucose-derived carbon among glycolysis, the tricarboxylic acid cycle, and the pentose phosphate pathway [34].

Third, JC-1, total cellular ATP, and ROS are indirect functional indicators. The present study did not directly assess mitochondrial abundance, respiratory function, ultrastructure, or network morphology. Consequently, the observed changes cannot be conclusively interpreted as mitochondrial fragmentation or as a specific form of mitochondrial damage. Future studies could validate mitochondrial membrane potential using TMRM or TMRE in combination with an FCCP control. MitoTracker or TOM20-based confocal imaging could be used to quantify mitochondrial length, branching, and fragmentation. Transmission electron microscopy, mitochondrial DNA copy number, and citrate synthase activity could also be used to evaluate mitochondrial abundance and structure. In addition, mitochondrial-specific ROS, oxidative damage products, and antioxidant enzyme activities should be measured to distinguish physiological ROS signaling from pathological oxidative stress [35].

Most importantly, the present study did not comprehensively examine phosphorylated AMPK, total AMPK, phosphorylated ACC, or the key phosphorylation sites of DRP1. Nor were AMPK- or DRP1-targeted intervention and rescue experiments performed. Therefore, the current results cannot demonstrate that PFKM regulates mitochondrial status and myogenic differentiation through the AMPK–DRP1 signaling axis. Previous studies have shown that energy stress can induce AMPK-mediated phosphorylation of MFF and thereby regulate DRP1-dependent mitochondrial fission [32]. However, whether this mechanism applies to porcine SMSCs with abnormal PFKM expression requires direct experimental verification. Future studies should measure the ratios of p-AMPK to AMPK and p-ACC to ACC, DRP1 phosphorylation at Ser616 and Ser637, and the recruitment of DRP1 to mitochondria. AMPK activators, inhibitors, or genetic interventions should then be used to determine whether the metabolic, mitochondrial, and differentiation phenotypes can be restored. Moreover, the myotube fusion index, myotube diameter, and number of myonuclei should be evaluated. Different PFKM expression levels and intervention time points should also be established to determine whether an optimal PFKM expression range exists for maintaining metabolic homeostasis and myogenic differentiation in SMSCs.

In conclusion, using a research strategy consisting of proteomic screening, candidate gene identification, and cellular functional validation, this study preliminarily demonstrated that dysregulated PFKM expression is closely associated with alterations in glycolysis-related metabolism, energy and redox homeostasis, mitochondrial function-related parameters, and myogenic differentiation capacity in porcine skeletal muscle satellite cells. The available data support PFKM as a candidate regulator of skeletal muscle metabolism and differentiation that warrants further investigation. However, they are insufficient to establish a definitive causal mechanism mediated by the AMPK–DRP1 pathway. This study provides a basis for subsequent investigations of metabolic flux, mitochondrial morphology, signaling pathways, and in vivo function, and offers preliminary evidence for the identification of candidate genes related to porcine meat production traits and for studies of gene regulation in genetic breeding.

## Data Availability

The original contributions presented in this study are included in the article/Supplementary Material. Further inquiries can be directed to the corresponding author.

## Supplementary Materials

The following supporting information can be downloaded at: https://www.mdpi.com/article/10.3390/vetsci13080742/s1, File S1: The Original WB Figures. (Full-length uncropped western blot images. Molecular weight markers (kDa) are labeled on the side of each blot).
