# Supplementary material for: PFKM Modulates Porcine Skeletal Muscle Satellite Cell Differentiation Through Metabolic and Mitochondrial Pathways
Source: Vet Sci. 2026 Jul 26;13(8):742. doi: 10.3390/vetsci13080742 (PMC13517790; doi:10.3390/vetsci13080742)
Supplement: Supplementary file 1 [file vetsci-13-00742-s001.zip › vetsci-4395764-supplementary File S1.pdf]

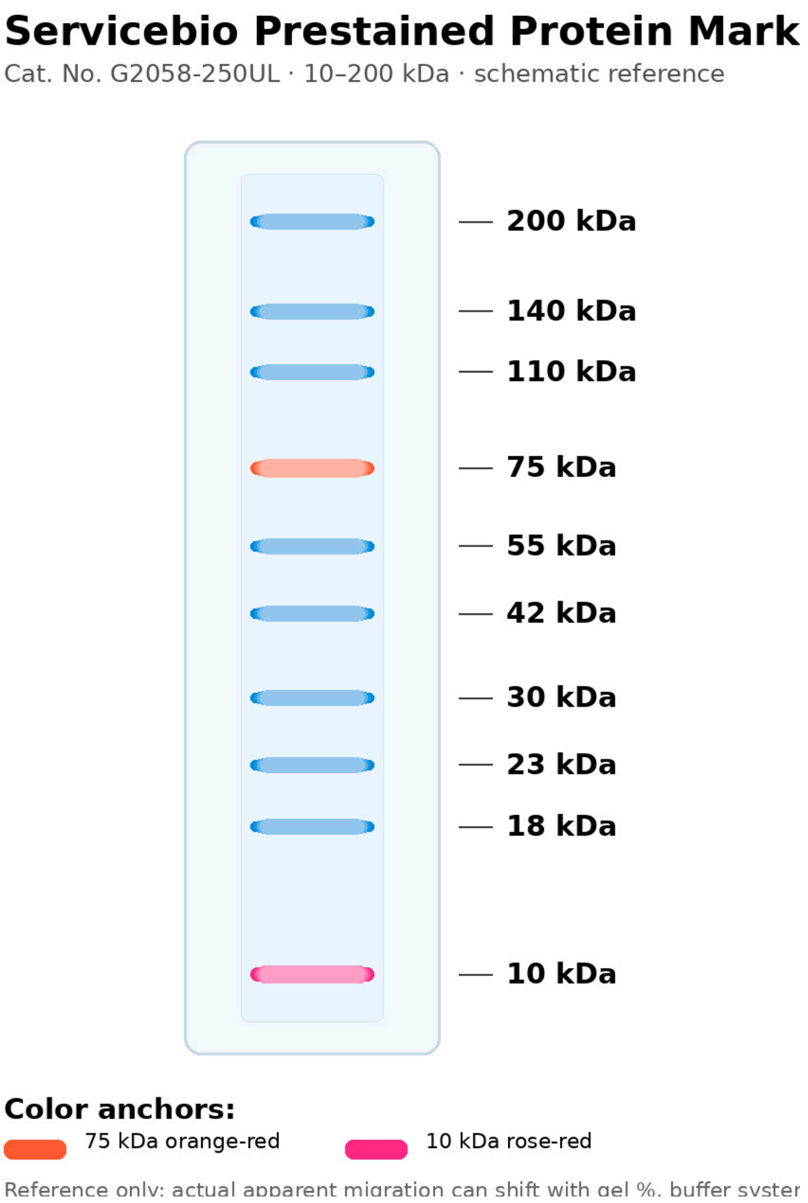

Prestained Protein Marker II (10–200 kDa; Cat. No. G2058, Servicebio, Wuhan, China) was used as the protein molecular weight marker for all SDS-PAGE and Western blot analyses.

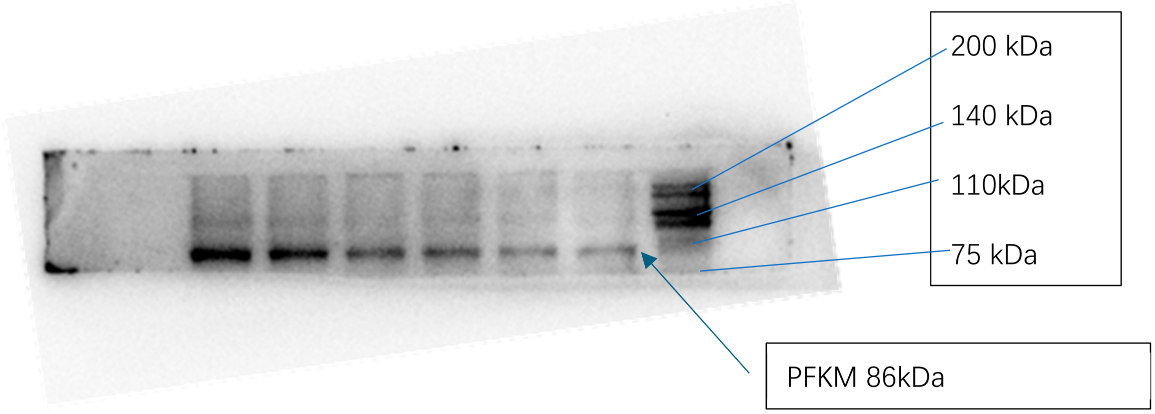

PFKM

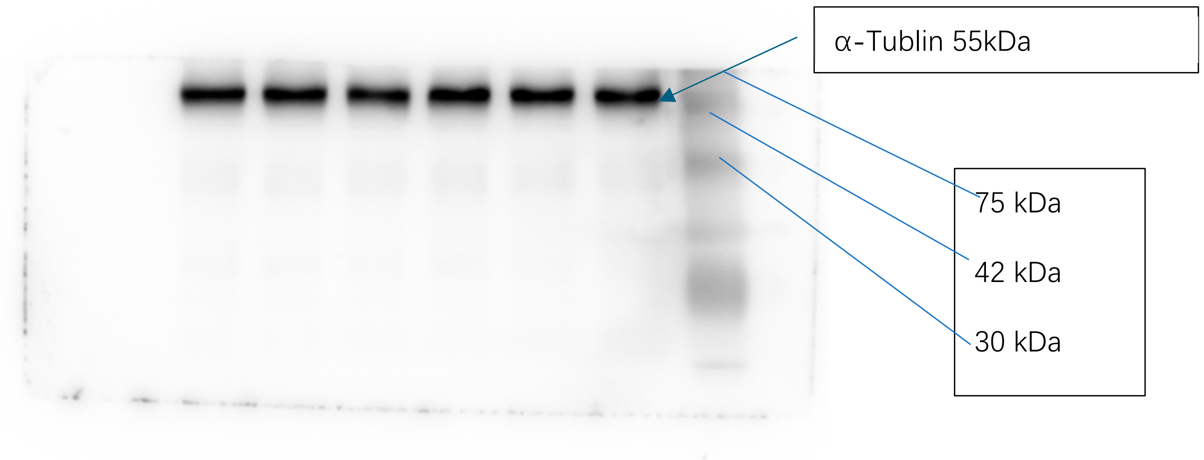

α-Tublin PFKM FOR Fig.1

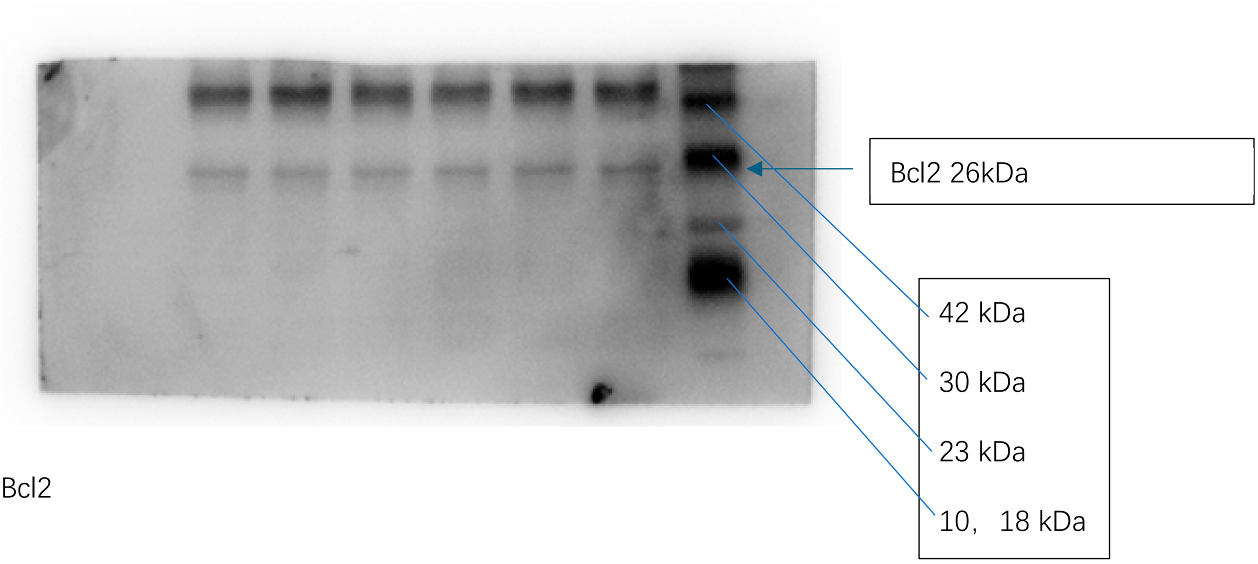

Bcl2

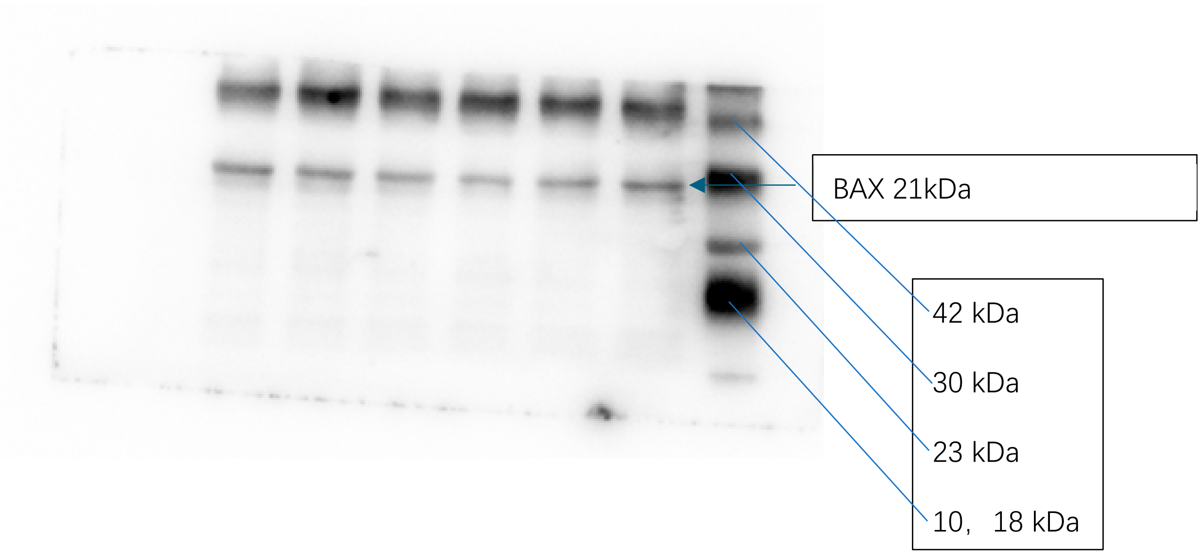

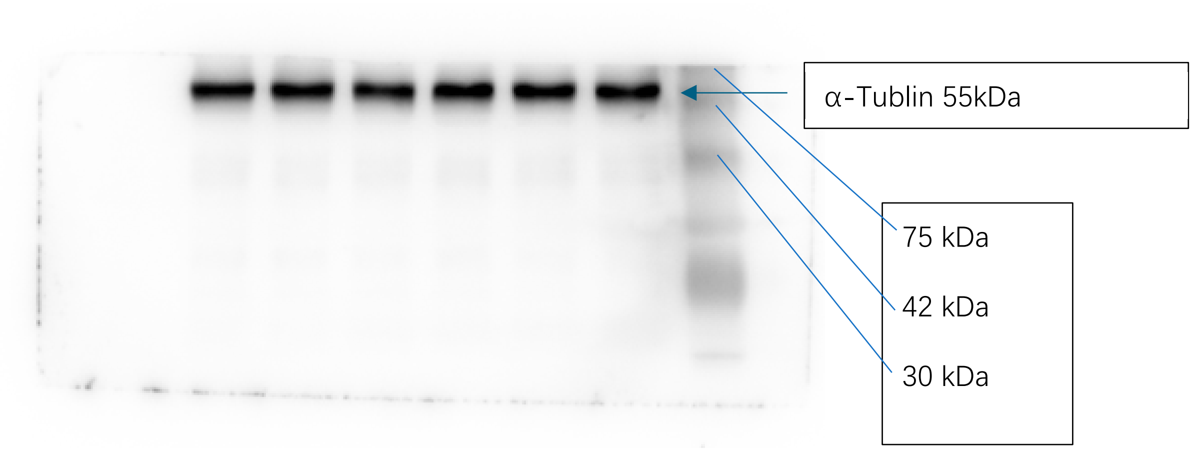

α-Tublin Bcl2 Bax FOR Fig.3

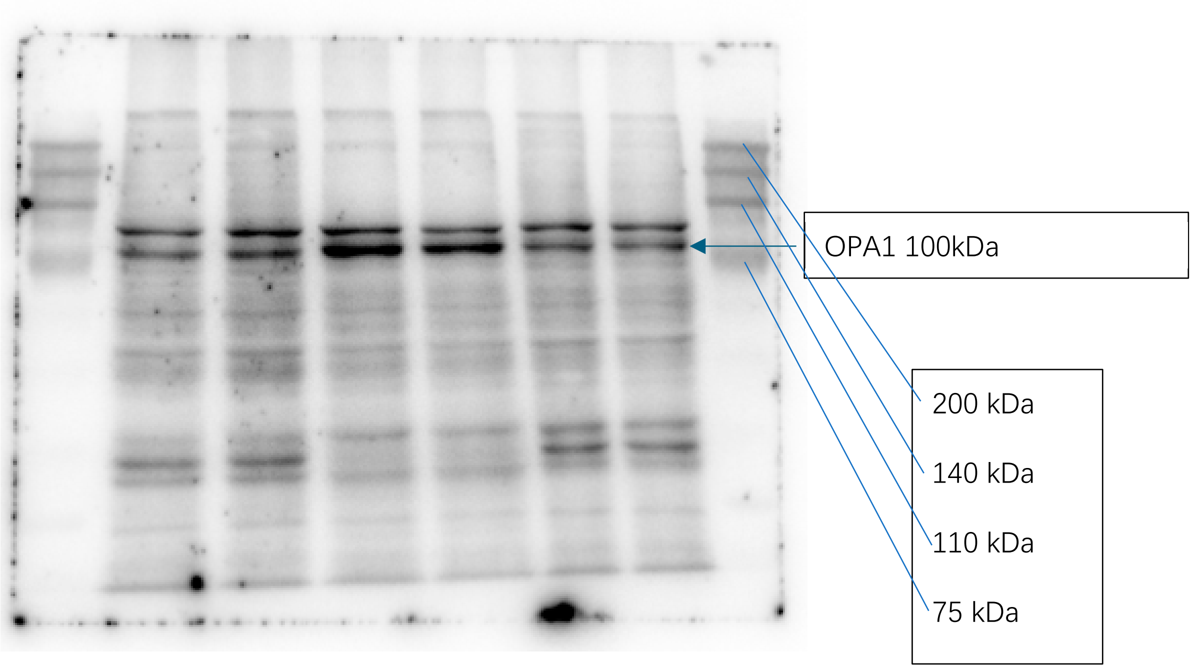

M

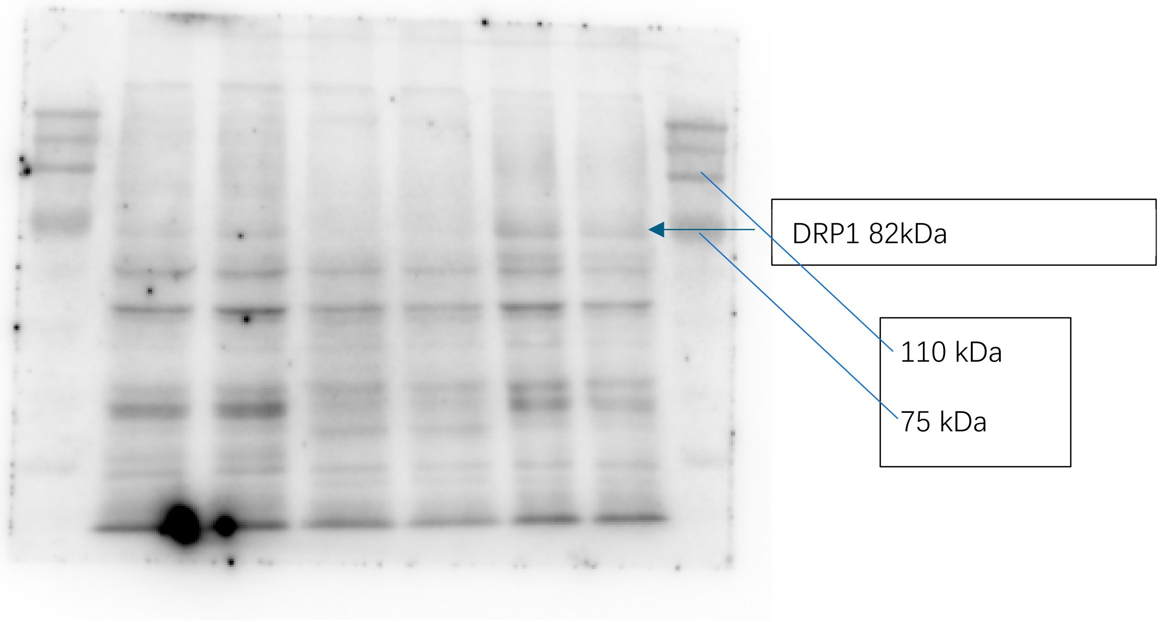
DRP1 82kDa
110 kDa
75 kDa

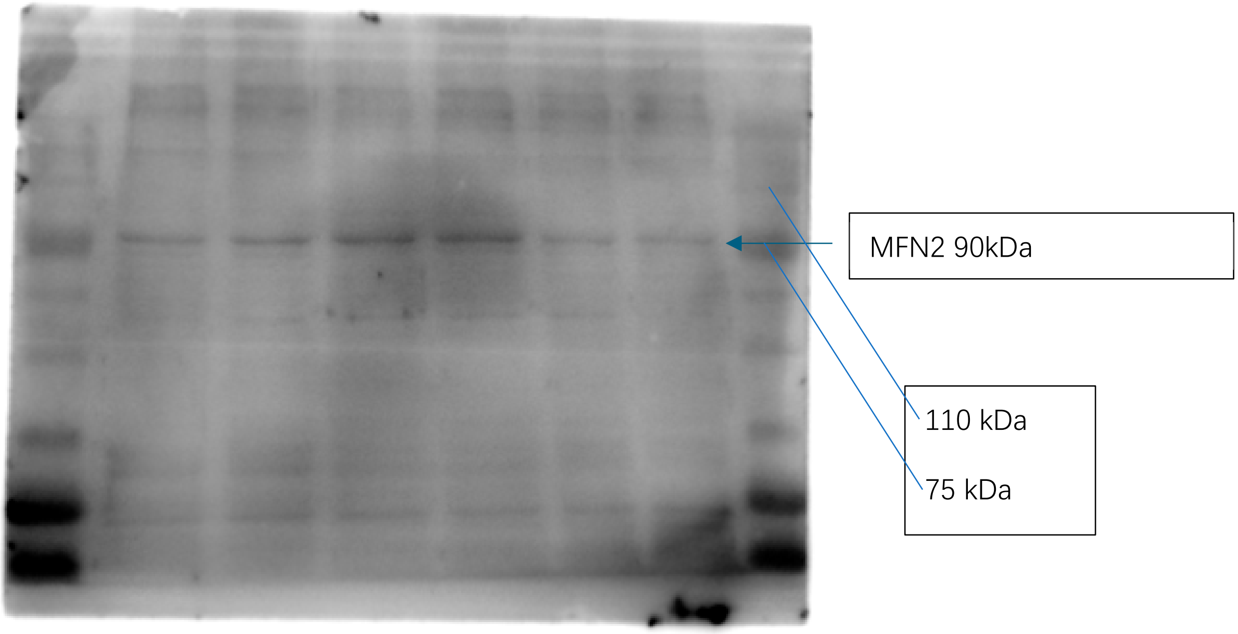
MFN2 90kDa
110 kDa
75 kDa

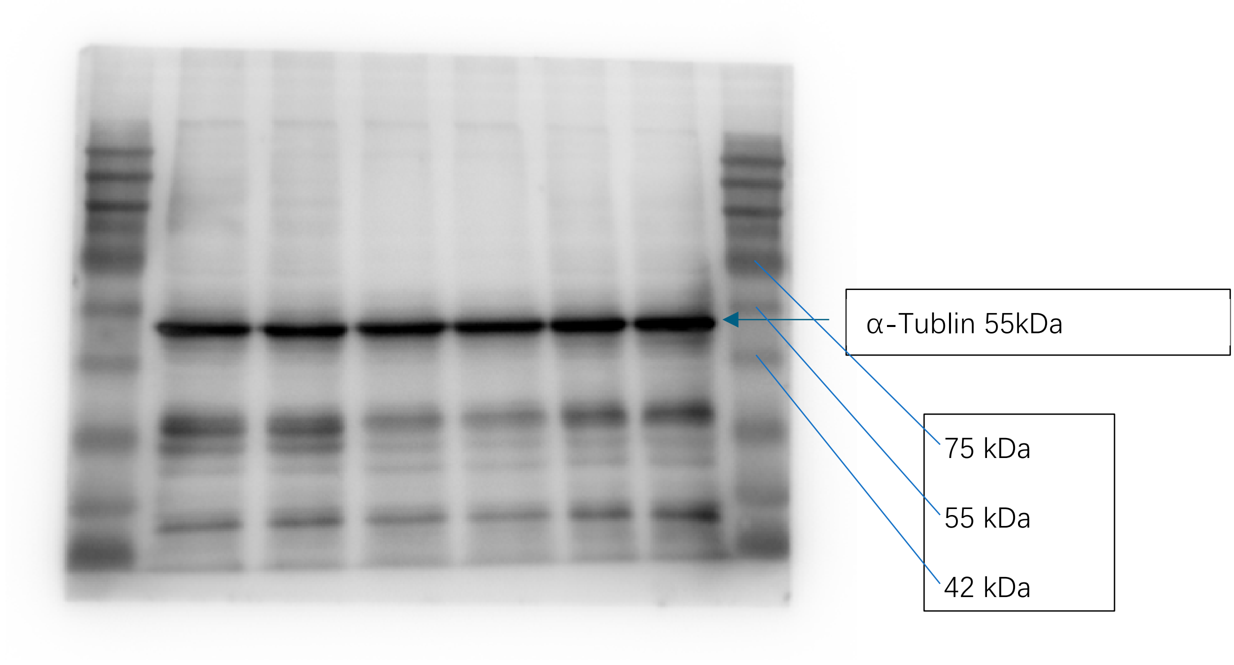

OPA1 DRP1 MFN2 α-Tublin FOR Fig.7

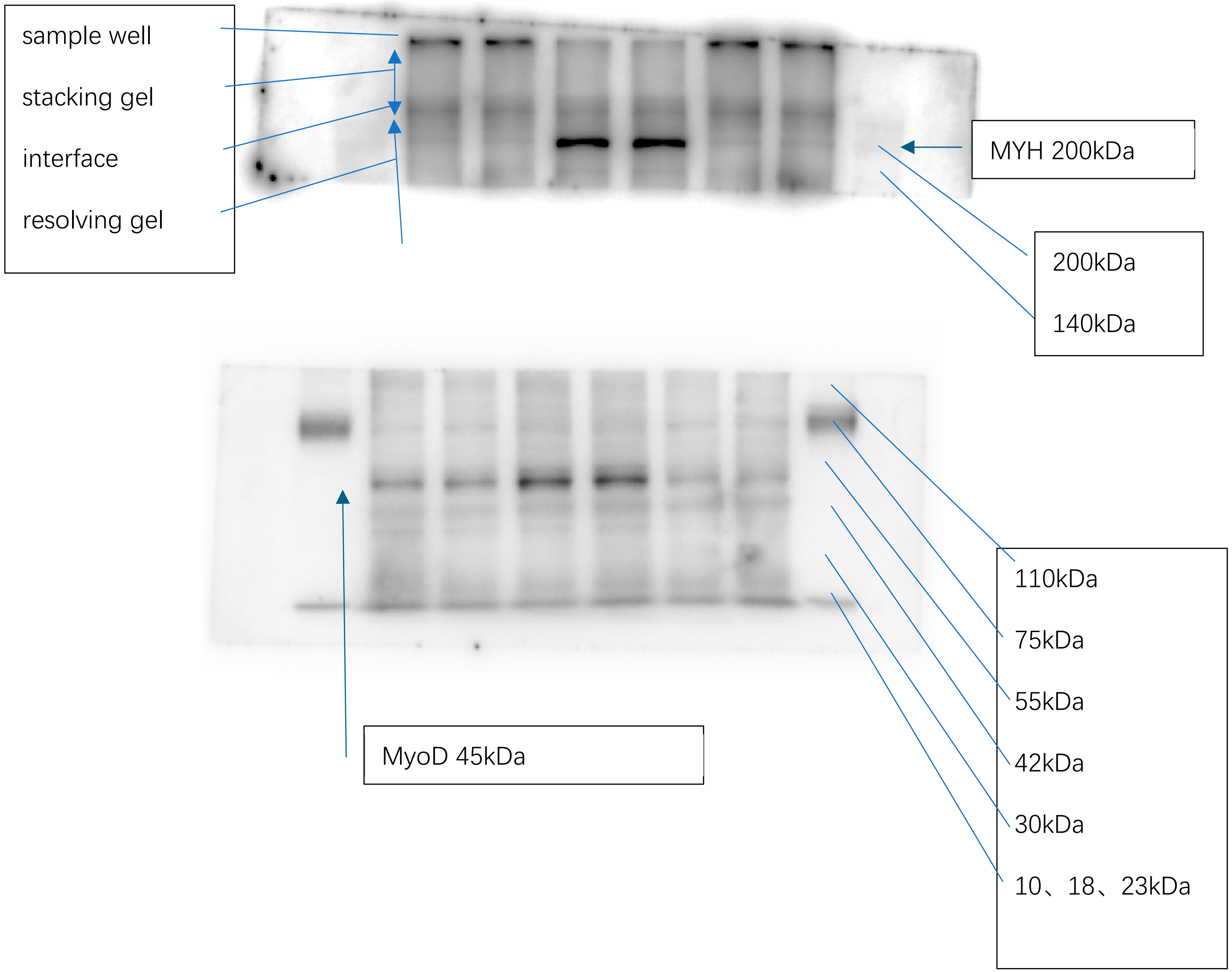
sample well
stacking gel
interface
resolving gel
MYH 200kDa
200kDa
140kDa
MyoD 45kDa
110kDa
75kDa
55kDa
42kDa
30kDa
10、18、23kDa

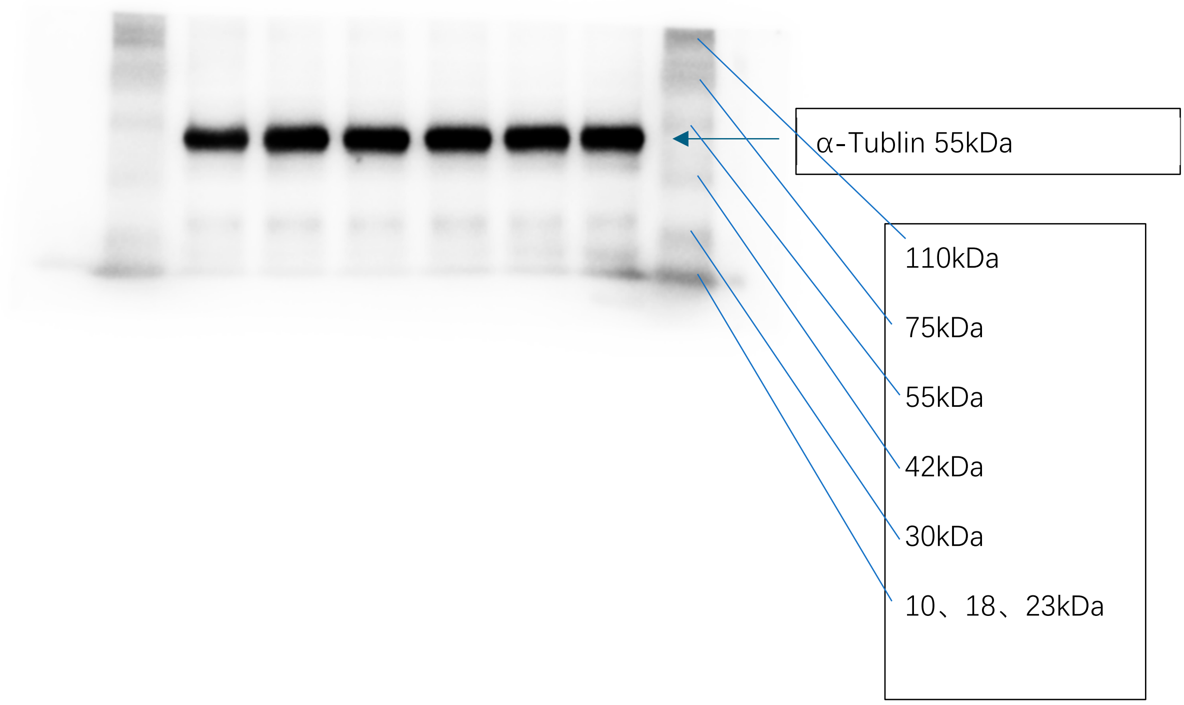

MyoD MYH α-Tublin FOR Fig.8
